# Global Insight into Lysine Acetylation Events and Their Links to Biological Aspects in *Beauveria bassiana*, a Fungal Insect Pathogen

**DOI:** 10.1038/srep44360

**Published:** 2017-03-15

**Authors:** Zhi-Kang Wang, Qing Cai, Jin Liu, Sheng-Hua Ying, Ming-Guang Feng

**Affiliations:** 1 Institute of Microbiology, College of Life Sciences, Zhejiang University, Hangzhou, 310058, China; 2 Shandong Provincial Key Laboratory of Microbial Engineering, Qilu University of Technology, Jinan, Shandong, 250353, China

## Abstract

Lysine acetylation (Kac) events in filamentous fungi are poorly explored. Here we show a lysine acetylome generated by LC-MS/MS analysis of immunoaffinity-based Kac peptides from normal hyphal cells of *Beauveria bassiana*, a fungal entomopathogen. The acetylome comprised 283 Kac proteins and 464 Kac sites. These proteins were enriched to eight molecular functions, 20 cellular components, 27 biological processes, 20 KEGG pathways and 12 subcellular localizations. All Kac sites were characterized as six Kac motifs, including a novel motif (KacW) for 26 Kac sites of 17 unknown proteins. Many Kac sites were predicted to be multifunctional, largely expanding the fungal Kac events. Biological importance of identified Kac sites was confirmed through functional analysis of Kac sites on Pmt1 and Pmt4, two *O*-mannosyltransferases. Singular site mutations (K88R and K482R) of Pmt1 resulted in impaired conidiation, attenuated virulence and decreased tolerance to oxidation and cell wall perturbation. These defects were close to or more severe than those caused by the deletion of *pmt1*. The Pmt4 K360R mutation facilitated colony growth under normal and stressful conditions and enhanced the fungal virulence. Our findings provide the first insight into the Kac events of *B. bassiana* and their links to the fungal potential against insect pests.

Lysine acetylation (Kac) controlled by lysine acetyltransferases is a dynamic and reversible process of post-translational modification (PTM) across organisms^1^,^2^. Such PTM processes are known to regulate gene expression via histone modification and is involved in the mediation of cellular metabolism^3^,^4^,^5^,^6^,^7^, protein folding^8^, sister chromatid cohesion^9^, and energy utilization^10^.

Advances in proteomic technology, such as liquid chromatography coupled with tandem mass spectrometry (LC-MS/MS), have facilitated characterization of PTM sites^11^,^12^,^13^,^14^,^15^. The recognized PTM sites help to understand their functional relevance and provide an overview of regulatory scope through a given PTM process. The generated PTM datasets are important resources for the *in silico* analyses to reveal biological properties and evolutionary trajectories of PTM sites. To date, LC-MS/MS-based acetylomic analyses have unveiled vital roles of Kac events in prokayotes, such as *Thermus thermophilus*^16^, *Escherichia coli*^17^,^18^, *Salmonella enterica*^19^, *Bacillus subtilis*^20^, *Mycobacterium tuberculosis*^21^, *Vibrio parahemolyticus*^22^, and *Streptomyces roseosporus*^23^. Eukaryotic acetylomic studies have also revealed different numbers of Kac events in *Saccharomyces cerevisiae*^24^, *Candida albicans*^25^, *Phytophthora sojae*^26^, *Arabidopsis thaliana*^27^,^28^, *Triticum aestivum*^29^, *Vitis vinifera*^30^, *Toxoplasma gondii*^31^, *Bombyx mori*^32^, *Drosophila melanogaster*^33^, *Mus musculus*^34^, *Rattus norvegicus*^35^ and human cells^13^,^35^,^36^,^37^. All of these studies have demonstrated that Kac is a ubiquitous PTM process linked to various cellular events in both prokaryotes and eukaryotes.

However, Kac events have not been well explored in filamentous fungi, which comprise many pathogens of plants, arthropods, animals and human. Among those, filamentous fungal insect pathogens, such as *Beauveria* and *Metarizhium* species, are important fungal agents for biological control of arthropod pests threatening agriculture, forestry and human health^38^,^39^. Aerial conidia and submerged blastospores of these insect pathogens are often used as active ingredients of fungal insecticides. Their biological control potential depends not only on their ability to infect target pests and adapt to diverse pest habitats but also on their proper development, which controls quantity and quality of conidia in mass production *in vitro* and propagation of blastospores (hyphal bodies) *in vivo* after entry into host haemocoel. All of fungal responses to host and environment are likely relevant to various cellular events under PTM control. Our recent analyses of quantitative proteome and phosphoproteome have revealed a hub role for the dual-specificity phosphatase Cdc14 in the signaling network that regulates cytokinesis, asexual development, multiple stress tolerance and virulence in *Beauveria bassiana*^40^, a classic insect pathogen that occurs in asexual cycle *in vitro* or *in vivo* and attacks the broadest spectrum of hosts among all fungal pathogens known to date^41^. In this study, we characterized Kac events in *B. bassiana* under normal conditions by constructing and analyzing the fungal lysine acetylome and found 462 Kac sites on 283 acetylated proteins falling into functionally diverse groups. Among the identified Kac sites, three on Pmt1 and Pmt4, two *O*-mannosyltransferases acting as regulators of vegetative growth, conidiation capacity, multiple stress tolerance and virulence in *B. bassiana*^42^, were functionally analyzed through site-direction mutation. The results provide the first insight into the links of Kac events to biological and cellular aspects of the fungal insect pathogen and its control potential against arthropod pests.

## Results

### Generation and analysis of *B. bassiana* lysine acetylome

**Figure 1:**
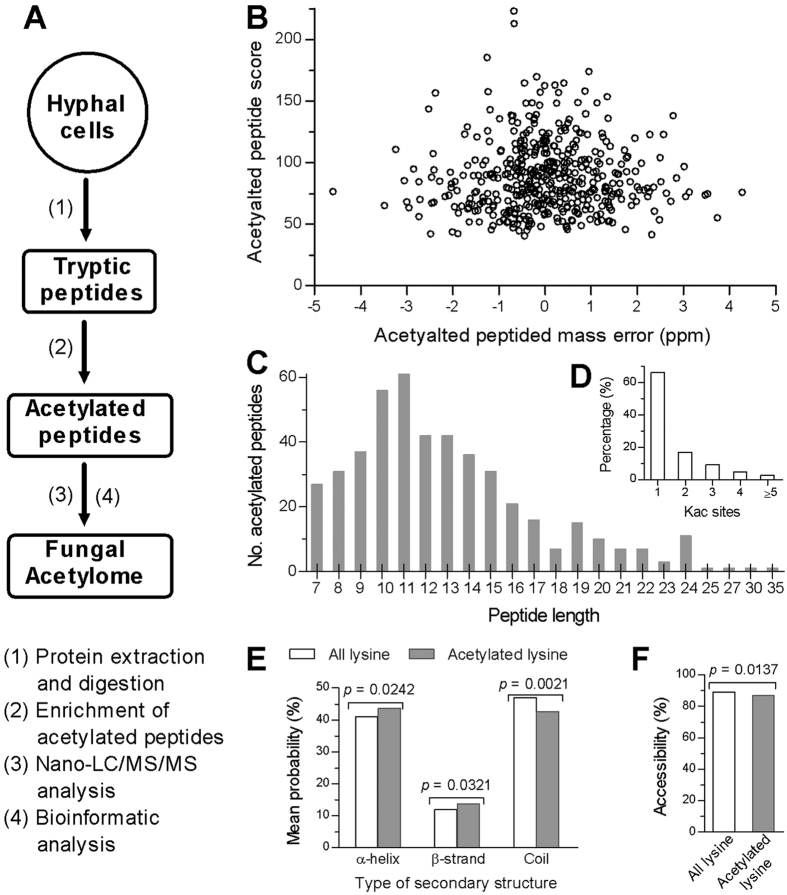
Outline of *B. bassiana* acetylome. (**A**) Workflow for creation of the acetylome through digestion of proteins with trypsin and enrichment of acetylated peptides, nano-LC/MS/MS analysis and bioinformatic annotation. (**B**,**C**) Distributions of mass errors and lengths for all identified Kac peptides. (**D**) Percentages of Kac proteins vectoring 1, 2, 3, 4, and ≥5 Kac sites, respectively. (**E**) Mean probabilities of lysine acetylation predicted for different types of protein secondary structures (α-helix, β-strand and coil). (**F**) Predicted surface accessibility of acetylated sites. The marked *p* values denote a significance of the non-paired Wilcox test for the predicted values in (**E,F**).

The lysine acetylome of *B. bassiana* was generated through purification and digestion of protein sample, affinity enrichment and purification of lysine acetylated peptides, nano-HPLC/MS/MS analysis and separation of lysine acetylated peptides, and annotation of lysine acetylated proteins (Fig. 1A). The mass errors of most acetylated peptides ranged from −3 to 3 ppm (Fig. 1B), satisfying an expected error control for the MS dataset. Most of the acetylated peptides fell in a length range of 8–20 amino acids (Fig. 1C), well in agreement with the property of tryptic peptides and the standard for preparation of protein sample. The fungal acetylome comprised 464 identified Kac sites on 283 Kac proteins (detailed in Table S1). The proportions of the Kac proteins harboring one, two, three, four and five or more Kac sites were 66%, 16.96%, 9.19%, 4.95% and 2.83% respectively, giving an average of 1.64 Kac sites per protein (Fig. 1D). There were 43.6%, 13.8% and 42.6% of the identified Kac proteins which were predicted to possess the secondary structures of α-helix, β-strand and coil respectively (Fig. 1E). Surface accessibility analysis demonstrated that 89.1% of all lysine residues and 86.8% of acetylated residues were localized to the protein surface, respectively (Fig. 1F).

### Functional annotation and enrichment analysis of *B. bassiana* Kac proteins

All of the identified Kac proteins were annotated with the predicted features of GO terms, subcellular localizations, motif logos, and FunCat and KOG classifications. Some of the Kac proteins identified from the acetylome were functionally diverse. As a result of the GO analysis, the Kac proteins enriched in the Biological Process category were linked to metabolic (63.25%) and cellular (49.82%) processes (detailed in Table S2) respectively. Classified to the Molecular Function category were catalytic enzymes (52.3%) and binding proteins (47.7%). The Cellular Component category comprised cell (35.7%), organelle (25.5%), macromolecular complex (23.4%) and membrane (14.5%). Different proportions of the Kac proteins with one or more Kac sites were predicted to localize mainly in cytoplasm (37.1%), mitochondria (24.0%), extracellular space (14.1%), nucleus (11.3%) and plasma membrane (5.3%), respectively (detailed in Table S3).

Noticeably, 27.21% and 18.02% of the Kac proteins in the Biological Process and Molecular Function categories were unclassified through the GO analysis. Further revealed by FunCat annotation, the identified Kac proteins were involved in protein binding (22.1%), metabolism (17.6%), cell rescue/defense/virulence (8.0%), energy (7.4%), protein synthesis (7.1%), cell cycle and DNA processing (3.9%), transcription (3.9%), environmental interaction (3.9%), cell fate (2.9%) and several other functions (detailed in Table S4). The KOG analysis unveiled the links of most Kac proteins to metabolism (41.6%), cellular processes and signaling (28.2%), and information storage and processing (19.6%), as detailed in Table S5.

**Figure 2:**
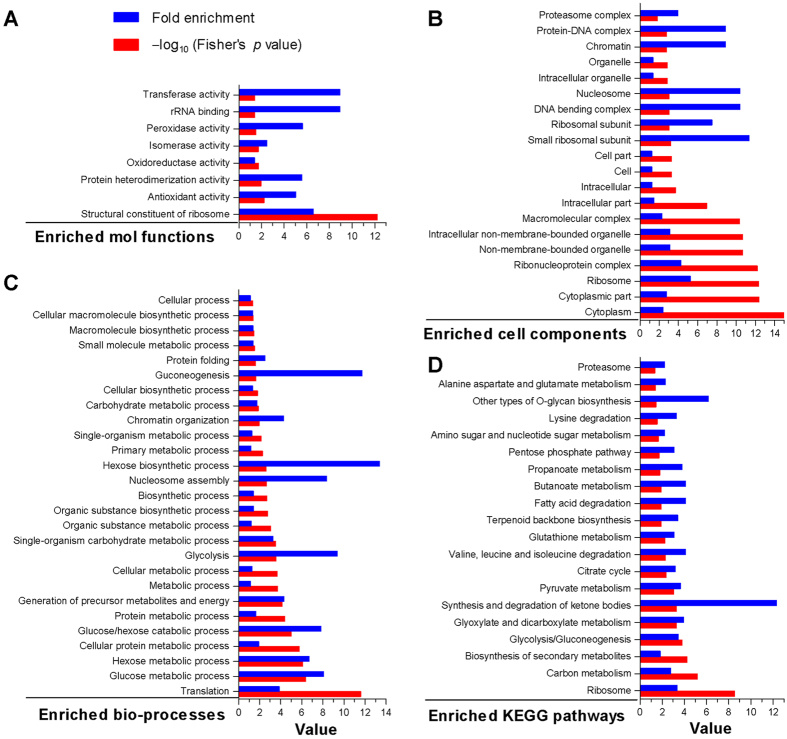
Features of *B. bassiana* acetylome. (**A–D**) Fold enrichment and Fisher’s *p* values for the Kac proteins enriched to molecular functions, cellular components, biological processes and KEGG pathways through GO and KEGG analyses, respectively.

In enrichment analysis, the identified Kac proteins were functionally involved in structural constituent of ribosome, protein heterodimerization activity, rRNA binding, and activities of transferases, isomerases and antioxidant enzymes (Fig. 2A). All of them were significantly enriched to 20 cell components (Fig. 2B) and 27 biological processes, such as chromatin organization, translation, nucleosome assembly, glucose/hexose catabolic process, glycolysis, gluconeogenesis and hexose biosynthesis at very high levels of significance (Fig. 2C). They were also significantly enriched to 20 KEGG pathways, such as ribosome, carbon metabolism, cirtrate cycle and glycolysis/gluconeogenesis (Fig. 2D).

All enrichment analyses indicated deep involvements of Kac events in multiple cellular events and processes, particularly metabolism, transcription and translation. These results highlight a significance of lysine acetylation for the life of *B. bassiana*.

### Kac motifs identified from the Kac sites of *B. bassiana*

**Figure 3:**
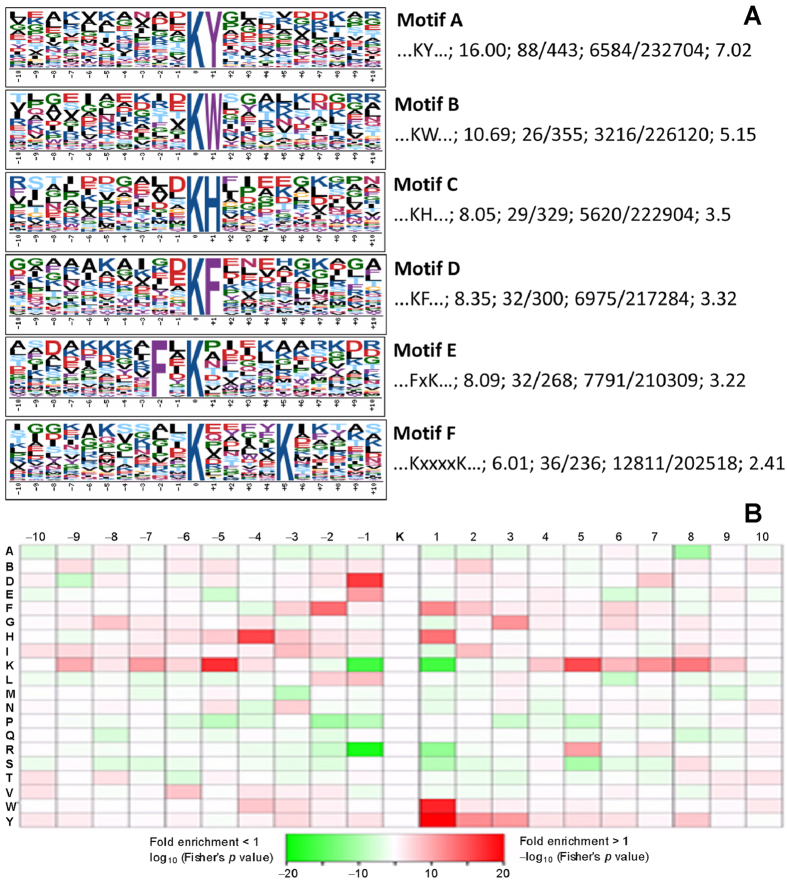
Kac motifs identified from the *B. bassiana* acetylome and their preferences to other amino acid residues. (**A**) Logos of six Kac motifs predicted with the WebLogo software at http://weblogo.berkeley.edu. The height of each amino acid indicates the level of conservation at that position. Each logo is followed by the outlined sequence of each motif, the motif score, the foreground counts of matches (F_match_)/identified Kac peptides (F_size_), the background counts of matches (B_match_)/all the identified peptides (B_size_) in the used database, and the fold increase, respectively. Fold increase = (F_size_/F_match_)/(B_size_/B_match_). (**B**) Enriched preferences of Kac sites to amino acid residues at particular positions. Red: significantly preferred residues. Green: not enriched around Kac sites.

Some amino acid residues have been shown to preferentially surround Kac sites in both eukaryotic and prokaryotic cells^16^,^17^,^34^,^43^. We predicted possible motifs involved in all of the identified Kac proteins using the Motif-x software designed to extract overrepresented patterns from any sequence set^44^. As a result, six conserved lysine acetylated motifs were predicted from 243 identified Kac sites at differentially enriched abundance, including KacY, KacW, KacH, KacF, FxKac and KacxxxxK (Fig. 3A). In these motifs, Kac denotes an acetylated lysine, and Y, W, H, F, K and x represent tyrosine, tryptophan, histidine, phenylalanine, lysine and random amino acid residue, respectively. As illustrated in Fig. 3B, Kac was present at the downstream positions of −5, −4, −2 and −1 for K, H, F and D residues or at the upstream positions of +5 for K or +1 for F, H, W and Y residues, respectively, in the predicted motifs. However, Kac did not occur in a preference to K at −1 and +1 positions and to R at −1 position. In other words, the six motifs showed the respective preferences of Kac to Y, W and H at +1 position, to K at +5, and to F at −2 or +1. Among the identified motifs, only KacW was enriched in the *B. bassiana* acetylome but was not found in the surveyed databases across organisms except the fungi *C. albicans*^25^ and *P. sojae*^26^. Other motifs were highly conserved because they appeared in the acetylomes of *E. coli*^9^,^17^, *V. parahemolyticus*^22^, *S. roseosporus*^23^, *Triticum aestivum*^29^, *V. vinifera*^30^, *B. mori*^32^, *D. melanogaster*^33^, *R. norvegicus*^35^ and/or human cells^34^,^45^.

### Interactions among Kac proteins of *B. bassiana*

**Figure 4:**
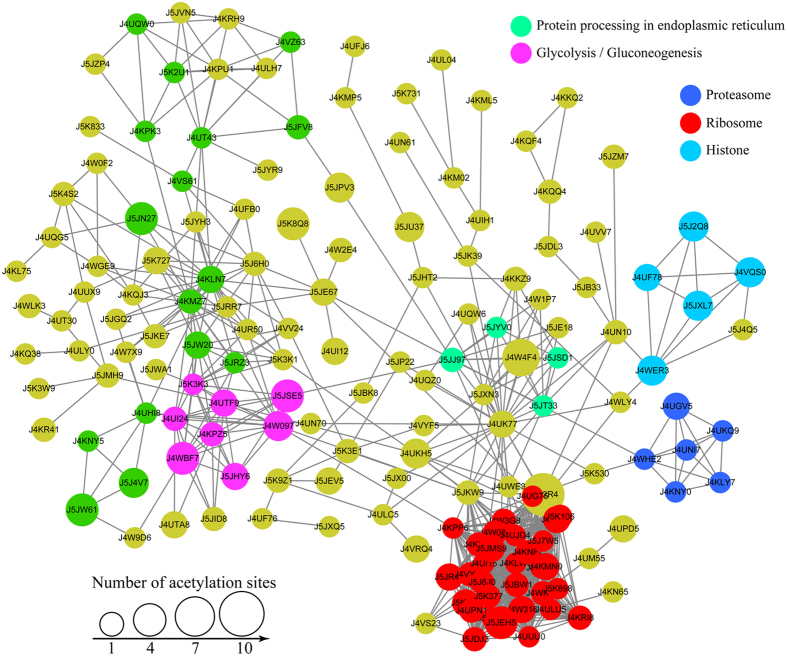
Interaction networks of Kac proteins in *B.bassiana*. Protein-to-protein interactions revealed by Cytoscape software occur among the top five clusters associated with ribosome (red), glycolysis/gluconeogenesis (pink), histone (blue), proteasome (dark blue), and protein processing in endoplasmic reticulum (green) respectively.

Half of the identified Kac proteins varying in cell component, molecular function, biological process, KEGG pathway and subcellular localization were predicted to involve in 10 clusters of protein-protein interaction networks in *B. bassiana* although another half could not be mapped to specific clusters. As illustrated in Fig. 4, the top five clusters comprised 31, 8, 6, 5 and 4 proteins associated with ribosome, glycolysis/gluconcongensis, proteasome, histone, and protein processing in endoplasmic reticulum, respectively. The interaction networks suggest that the Kac proteins involve in a variety of physiological processes and cellular events/functions essential for the fungal adaptation to host and environment.

### Biological importance of Kac events in *B. bassiana*

**Figure 5:**
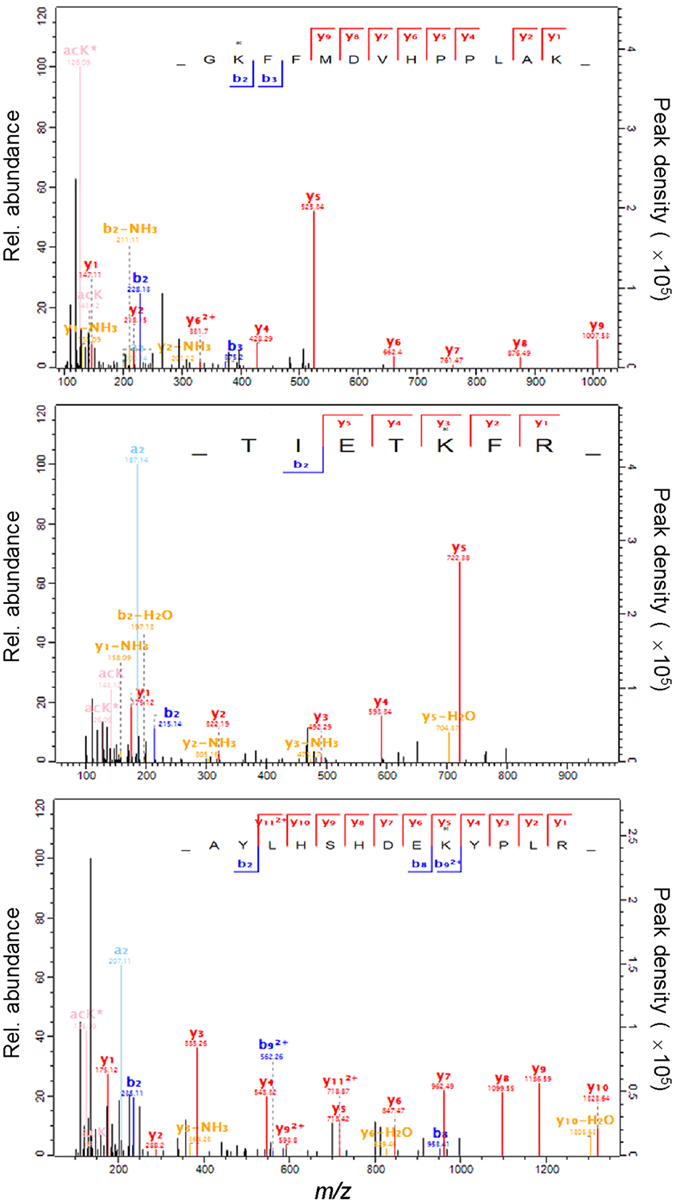
LC-MS/MS spectra for identification of Kac sites from Pmt1 and Pmt4 (*O*-mannosyltransferases) in the *B. bassiana* acetylome. Relative abundance (left Y-axis) and peak density (right Y-axis) of accumulated ions over the marked *m*/*z* range indicate that the acetylated residues are K88 (**A**) and K482 (**B**) on Pmt1 and K360 (**C**) on Pmt4.

**Figure 6:**
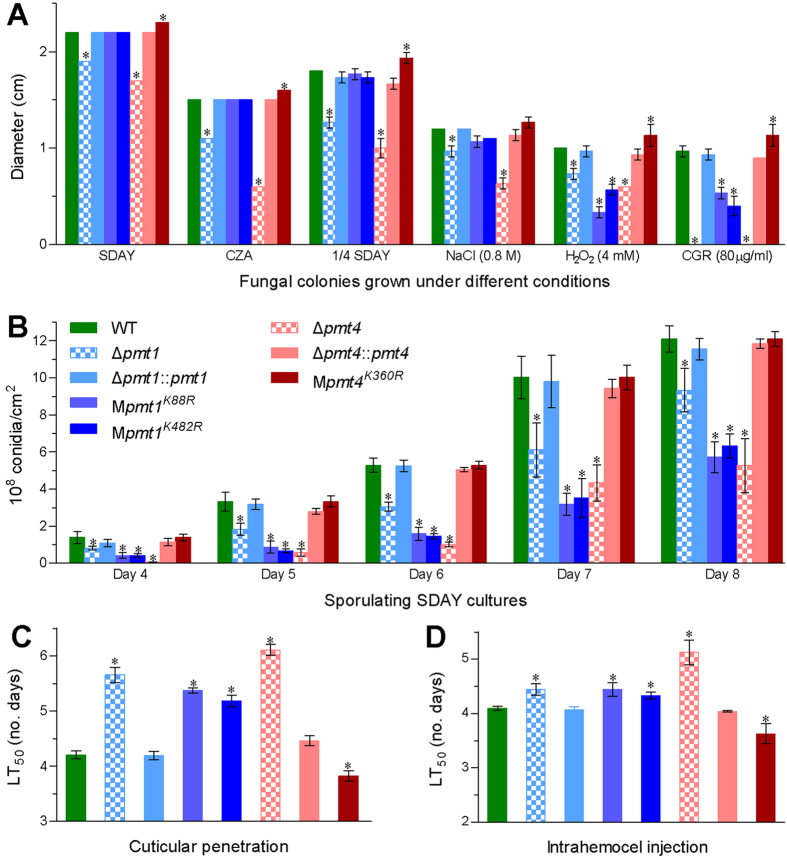
Phenotypes of *B. bassiana* altered by *pmt1* or *pmt4* deletion and singular Kac site mutations. (**A**) Diameters of fungal colonies after 7 days of incubation at 25 °C in rich SDAY, 1/4 SDAY and minimal CZA alone or in 1/4 SDAY supplemented with NaCl (0.8 M), H_2_O_2_ (4 mM) and CGR (Congo red 80 mg/ml) respectively. Each colony was initiated with 1 μl of a 10^6^ conidia/ml suspension. (**B**) Conidial yields quantified daily from the SDAY cultures over the days of incubation at 25 °C in a light/dark cycle of 12:12 h. All cultures were initiated by spreading 100 μl of a 10^7^ conidia/ml suspension per plate. (**C**,**D**) LT_50_ (no. days) for fungal virulence to *G. mellonella* larvae via cuticular penetration (immersed) and cuticle-bypassing infection (injected), respectively. Asterisked bars differ significantly from those unmarked in each group of bars within each chart (Tukey’s HSD, *p < *0.05). Error bars: SD from three replicates.

In the *B. bassiana* acetylome, K88, K482 and K360 were identified as Kac sites of the *O*-mannosyltransferases Pmt1 and Pmt4 respectively (Fig. 5). The three Kac sites were chosen to reveal biological links of the Kac sites identified from the acetylome by making use of the Δ*pmt1* and Δ*pmt4* mutants constructed previously^42^. As illustrated in Fig. 6A, the Pmt1 site-mutated strains M*pmt1*^*K88R*^ and M*pmt1*^*K482R*^ grew as well as wild-type (WT) and complemented strains on the plates of Sabouraud dextrose agar plus yeast extract (SDAY), 1/4 SDAY (amended with 1/4 of each SDAY nutrient) and Czapek agar (CZA) at 25 °C while M*pmt4*^*K360R*^ grew significantly faster than its control strains (Tukey’s HSD, *p* < 0.05). M*pmt1*^*K88R*^ and M*pmt1*^*K482R*^ were more sensitive to oxidative stress of H_2_O_2_ (4 mM), but much less sensitive to cell wall stress of Congo red (80 μg/ml), than Δ*pmt1* during cultivation in 1/4 SDAY. Both H_2_O_2_ and Congo red facilitated significantly the growth of M*pmt4*^*K360R*^ but suppressed the growth of Δ*pmt4*. However, all of the three Kac site-mutated strains showed null response to osmotic stress of 0.8 M NaCl (Tukey’s HSD, *p* > 0.05) although the fungal osmosensitivity increased in the absence of *pmt1* or *pmt4*.

Intriguingly, conidiation capacity during optimal cultivation in SDAY was much more impaired by each Kac site mutation of Pmt1 than by the deletion of *pmt1* (Fig. 6B). The mean (±SD) of conidial yields quantified from the WT cultures reached 5.28 (±0.39), 10.02 (±1.15) and 12.10 (±0.71) × 10^8^ conidia/cm^2^ on days 6, 7 and 8 respectively. Compared with these quantities, conidial yields of M*pmt1*^*K88R*^ and M*pmt1*^*K482R*^ decreased by 70% and 73% on day 6, 68% and 65% on day 7, and 53% and 48% on day 8, respectively, while the yield decrease in the Δ*pmt1* culture diminished to 42% on day 6, 39% on day 7 and 23% and day 8. However, conidial yield was not affected in M*pmt4*^*K360R*^ compared with a significant reduction in Δ*pmt4*.

In standardized bioassays, median lethal time (LT_50_) for the WT against *Galleria mellonella* larvae was 4.2 (±0.07) days through the normal route of cuticle infection (Fig. 6C) and 4.1 (±0.04) days through cuticle-bypassing infection (Fig. 6D). The LT_50_s for the mutants Δ*pmt1*, M*pmt1*^*K88R*^ and M*pmt1*^*K482R*^ against the model insect increased by 34%, 28% and 23% as compared with the WT estimate via the normal infection, respectively, and also elongated similarly at lesser level via the cuticle-bypassing infection. In contrast, the lethal action was significantly accelerated in M*pmt4*^*K360R*^ but delayed in Δ*pmt4* versus the WT irrespective of the cuticle or cuticle-bypassing infection.

These experimental data indicated that all of the Kac sites were crucial for Pmt1 and Pmt2 to sustain vegetative growth, aerial conidiation, stress responses and/or virulence of *B. bassiana*.

## Discussion

Our acetylomic analysis revealed 283 Kac proteins and 464 Kac sites in *B. bassiana*. The proportion of the identified Kac proteins (2.73%) in the fungal database of 10,364 proteins is relatively small compared with what was reported from the acetylome of *S. cerevisiae* (17.93%)^24^ but close to those in the aceylomes of *C. albicans* (5.28%)^25^ and *P. sojae* (4.34%)^26^. These fungal acetylomes are based on different genetic backgrounds and life styles, which are represented by the *B. bassiana* strain lacking sexual type, the lysine auxotroph strain and the lysine auxotroph *rpd3*-deletion strain (Δ*rpd3*) of *S. cerevisiae*, and the *C. albicans* and *P. sojae* strains with sexual and asexual types. The *S. cerevisiae* acetylome containing much greater proportion of Kac proteins than those of three other fungi implicates that proteins could be hyperacetylated in the used yeast strains. An expanded survey of reported lysine acetylomes from different organisms demonstrates a proportion of Kac proteins ranging from 0.12% to 11.73% in the protein databases of several representative eukaryotes except the yeast and from 1.85% to 42.83% in various bacteria, as summarized in Table 1. Indeed, the proportion of the Kac proteins identified from the acetylome of *B. bassiana* is higher than those in most acetylomes of other eukaryotes.

Kac events are well outlined by Kac motifs. Previous acetylomic studies have revealed several Kac motifs more or less varying with organisms and preferential locations of their Kac sites adjacent to Y, H, F, and K residues^16^,^17^,^34^,^43^. In this study, we identified six Kac motifs and found that these Kac sites preferred to locate upstream of Y, W, H, F and K residues (KacY, KacW, KacH, KacF and KacxxxxK) or downstream of F residue (FxKac) in *B. bassiana*. Five of these motifs also exist in the Kac proteins of prokaryotes and other eukaryotes and hence are highly conserved across organisms. The evolutionarily conserved traits of these motifs are often seen in some key Kac proteins and their Kac sites. For instance, paired Kac sites on the 14-3-3 proteins Bmh1 (K68 and K122) and Bmh2 (K70 and K124) in *B. bassiana* are well aligned with those (K70 and K125 on Bmh1 or K70 and K124 in Bmh2) in *S. cerevisiae*. Intriguingly, the KacW motif was characterized from 26 Kac sites of 17 uncharacterized Kac proteins in the *B. bassiana* acetylome and also found in the acetylomes of *C. albicans*^25^ and *P. sojae*^26^. This indicates that at least some Kac events are of special importance for the fungal pathogens and perhaps more fungi whose lysine acetylomes have not been explored.

Moreover, many of the Kac proteins with one or more Kac sites are multifunctional, expanding largely the links of Kac events to biological aspects in *B. bassiana*. The fungal Kac events are distributed in all possible intracellular parts based on predicted subcellular locations and hence are enriched in plenty of molecular functions, biological processes and KEGG pathways at transcriptional and/or posttranscriptional levels. The biological importance of the identified Kac events was verified by functional analysis of the Kac sites on Pmt1 and Pmt2. Particularly, expression of the site-mutated allele *pmt1*^*K88R*^ or *pmt1*^*K482R*^ in Δ*pmt1* resulted in severe defects in conidiation, virulence and tolerance to oxidation and cell wall perturbation. These defects are close to or even more severe than those in the absence of *pmt1*. This highlights an essentiality of either K88 or K482 for a significant role of Pmt1 in the asexual development, host infection and some stress responses of *B. bassiana*. Intriguingly, the *pmt4*^*K360R*^ mutation facilitated the fungal growth under normal conditions and the stresses and enhanced virulence irrespective of cuticle or cuticle-bypassing infection. These changes also indicate a significance of K360 for the function of Pmt4 in *B. bassiana*. Aside from the two *O*-mannosyltransferases, several other Kac proteins identified in this study have also been shown to function diversely in *B. bassiana*, such as Bmh1 and Bmh2, the Ras GTPase Ras1 and the mitogen-activated protein kinase Hog1. Of those, Bmh1 and Bmh2 are essential for cell cycle, carbon/nitrogen utilization, conidiation, germination, multiple stress responses and host infection^46^. Ras1 is evidently involved in regulating germination, cell differentiation, hyphal growth, conidiation, pathogenicity and multiple stress responses^47^. Hog1 regulate positively cellular responses to hyperosmotic, oxidative and thermal stresses and negatively the fungal resistance to fludioxonil^48^. Noticeably, the mentioned proteins harbor not only the Kac sites identified in this study but also conserved phosphorylation sites, as revealed previously in our phosphoproteomic analysis^40^. The identified Kac proteins may also harbor some other PTM sites. It remains unclear what part the lysine acetylation takes in the multiple functions of the mentioned proteins. Nevertheless, the biological importance of the Kac sites identified in this study was revealed through functional analysis of the Kac sites on Pmt1 and Pmt4, thereby providing a useful database for further exploring possible roles of the Kac-linked cellular events in the life of *B. bassiana*.

## Methods

### Cultivation of microbial strains

The WT strain *B. bassiana* ARSEF2860 and its mutants were cultivated at 25 °C in a light/dark cycle of 12:12 h in rich SDAY (4% glucose, 1% peptone and 1.5% agar plus 1% yeast extract) for normal growth and development or in 1/4 SDAY and minimal CZA (3% sucrose, 0.3% NaNO_3_, 0.1% K_2_HPO_4_, 0.05% KCl, 0.05% MgSO_4_ and 0.001% FeSO_4_ plus 1.5% agar) for phenotypic experiments. *E. coli* DH5α from Invitrogen (Shanghai, China) was cultivated in Luria-Bertani medium at 37 °C for plasmid propagation.

### Preparation of protein samples

The WT strain was grown in cellophane-overlaid SDAY by spreading 100 μl of a 10^7^ conidia/ml suspension per plate, followed by 3 days of incubation at the optimum 25 °C. The resultant hyphal culture was ground in liquid nitrogen, suspended in a lysis buffer [8 M urea, 10 mM DTT (dithiothreitol), 2 mM EDTA (ethylenedinitrilo tetraacetic acid), 3 μM TSA (Trichostatin A), 50 mM NAM (nicotinamide) and 1% Protease Inhibitor Cocktail III (Sigma)] and sonicated three times on ice in a high-intensity ultrasonic processor (Scientz, Shanghai, China). After 10 min centrifugation by 20,000 *g* at 4 °C, the supernatant was discarded, and proteins were precipitated with cold 15% TCA (trichloroacetic acid) for 2 h at 4 °C, followed by washing with cold acetone three times. The precipitate was redissolved in a buffer (pH 8.0) of 8 M urea, and the protein concentration was determined with a 2-D Quant Kit (GE Healthcare Bioscience, Shanghai, China). The protein solution was reduced with 10 mM DTT for 1 h at 37 °C, alkylated with 20 mM IAA (iodoacetamide) for 45 min at room temperature in darkness, and then diluted with 100 mM NH_4_HCO_3_ until the urea concentration was lower than 2 M. The proteins in the solution were digested with trypsin at the trypsin-to-protein mass ratio of 1:50 for overnight and of 1:100 for another 4 h. The prepared protein sample was analyzed at Jingjie PTM Biolabs (Hangzhou) Co., Ltd. (Hangzhou, Zhejiang, China) as follows.

### Enrichment of Kac peptides

The digested protein sample was dissolved in NETN buffer (100 mM NaCl, 1 mM EDTA, 50 mM Tris-HCl and 0.5% NP-40, pH 8.0) and then incubated with pre-washed antibody beads (PTM Biolabs Inc., Chicago, IL, USA) at 4 °C overnight with gentle shaking. The beads were washed four times with the buffer and twice with dd-H_2_O. The bound peptides were eluted from the beads with 0.1% TFA (trifluoroacetic acid, Sigma-Aldrich). All of the eluted fractions were combined and vacuum-dried. The resulting Kac peptides were cleaned with C18 ZipTips (Millipore, Shanghai, China) following the manufacturer’s guide.

### LC-MS/MS analysis of Kac peptides

The cleaned Kac peptides dissolved in 0.1% FA (formic acid) were loaded onto the reversed-phase pre-column Acclaim PepMap 100 (Thermo Scientific). Peptide separation was performed using the reversed-phase analytical column Acclaim PepMap RSLC (Thermo Scientific). The gradient of solvent B (0.1% FA in 98% ACN) increased from 6% to 8% in 2 min, 8% to 22% in 20 min and 22% to 35% in 10 min respectively, climbed to 85% in 5 min, and then held at 85% for the last 3 min, all at a constant flow rate of 300 nl/min on an EASY-nLC 1000 UPLC system. The resulting peptides were analyzed in a Q Exactive^TM^ Hybrid Quadrupole-Orbitrap Mass Spectrometer (Thermo Scientific).

The Kac peptides were subjected to a NanoSpray Ionization (NSI) source, followed by LC-MS/MS scanning in Q Exactive^TM^ Plus coupled online to the UPLC. Intact peptides were detected in the orbitrap at a resolution of 70,000. The Kac peptides were selected with 28% NCE for MS/MS. Ion fragments in the orbitrap were detected at a resolution of 17,500. A data-dependent procedure alternated between one MS scan, followed by 20 MS/MS scans, to collect top 20 precursor ions for the Kac peptides above a threshold ion count of 2 × 10^4^ in the MS survey scan with 10 s dynamic exclusion at an electrospray voltage of 2.0 kV. Automatic gain control (AGC) was applied to prevent the ion trap from overfilling, resulting in accumulated 5 × 10^4^ ions for generation of MS/MS spectra. A scan range of 350 to 1800 *m*/*z* was applied for the MS scans. Three types of secondary structures (α-helix, β-strand and coil) were analyzed using NetSurfP software at http://www.cbs.dtu.dk/services/NetSurfP/. For each type, the mean probability of acetylated lysine residues was compared with that of all lysine residues in all acetylated proteins through non-paired Wilcox test.

All mass spectrometry proteomics data have been deposited to the ProteomeXchange Consortium via the PRIDE [1] partner repository with the dataset identifier PXD003682.

### Database search

The search engine MaxQuant (v.1.4.2) was used to process all MS/MS data. Tandem mass spectra were searched against the *B. bassiana* database^49^ from Uniport (10,366 sequences, Dec 2015) concatenated with a reverse decoy database. Trypsin/P was specified as a cleavage enzyme allowing for up to four missing cleavages, five modifications per peptide and five charges at the mass error level of 10 ppm for precursor ions and of 0.02 Da for fragment ions. Carbamidomethylation on Cys was specified as fixed modification, and oxidation on Met, acetylation on Lys and acetylation on protein N-terminus were specified as variable modifications. False discovery rate (FDR) thresholds for protein, peptide and modification site were specified at 1%. Minimal peptide length was set to seven residues. All of other parameters in the MaxQuant search were set to default values. The site localization probability was set to no less than 0.75.

### Bioinformatic annotation of Kac proteins

The Kac proteins identified through the above search were analyzed using several bioinformatic tools. Gene Ontology (GO) analysis was performed for classification of all identified acetylated proteins to three categories [cell component (GOCC), molecular function (GOMF) and biological process (GOBP)] by means of UniProt-GOA database at http://www.ebi.ac.uk/GOA/, InterProScan at http://www.ebi.ac.uk/interpro/ and GO annotation at http://geneontology.org/. WoLF PSORT program at http://wolfpsort.seq.cbrc.jp/ was used to predict if each identified Kac protein is localized to extracellular space (Extr), plasma membrane (Plas), cytoplasm (Cyto), nucleus (Nucl), mitochondria (Mito), endoplasmic reticulum (ER), peroxisome (Pero) or cytoskeleton (Cysk). Functional category (FunCat) annotation was carried out to predict intracellular roles and functions of all Kac proteins based on the PEDANT 3 database at http://pedant.gsf.de/. In Eukaryotic Orthologous Group (KOG) analysis, all sequences of the identified proteins were aligned with the KOG protein sequence database at http://genome.jgi.doe.gov/help/ kogbrowser.jsf by setting filter parameters to E value < 10^−5^, sequence identity >80%, and percent match length >60%. Conserved domain(s) of each Kac protein was predicted with InterProScan based on InterPro domain database at http://www.ebi.ac.uk/interpro/. Kyoto Encyclopedia of Genes and Genomes (KEGG) analysis at http://www.genome.jp/kegg/ was performed to annotate functional pathways of all identified Kac proteins. The online software Motif-x at http://motif-x.med.harvard.edu/ was used to predict motif sequences at specific Kac positions. Protein-protein interaction networks for the identified Kac proteins were analyzed with Cytoscape software^50^. In these analyses, all database protein sequences were used as the background database parameter, and other parameters were set to the default values. The method of Fisher’s exact test was used to gain enriched functional terms. An enriched Kac protein was considered to be significant if Fisher’s *p* < 0.05.

### Functional analysis of selected Kac sites

The Kac sites K88 and K482 on Pmt1 and K360 on Pmt4 found in the acetylome were mutated to the non-Kac residue Arg (R) by amplifying the full-length coding sequences and flanking regions of *pmt1*^K88R^, *pmt1*^K482R^, and *pmt4*^K360R^ with paired primers (Table S6) under the action of a KOD FX Neo DNA polymerase (Toyobo Co. Ltd., Osaka, Japan). The amplified sequences were digested with *Dpn*I, verified by sequencing at Invitrogen (Shanghai, China) and then inserted into the backbone plasmid p038-*sur*-gateway^51^ to exchange for the gateway fragment. The new plasmids with the site-mutated target genes were ectopically integrated into the Δ*pmt1* and Δ*pmt4* mutants as described previously^42^. Putative mutant colonies were screened in terms of their *sur* resistance to chlorimuron ethyl (10 μg/ml) in a selective medium, and then identified via PCR and quantitative real-time PCR (qRT-PCR) with paired primers (Table S6), as described elsewhere^42^,^51^. Positive site-mutated strains, i.e., M*pmt1*^*K88R*^, M*pmt1*^*K482R*^ and M*pmt4*^*K360R*^ identified via PCR and qRT-PCR (Fig. S1), were evaluated in parallel with Δ*pmt1*, Δ*pmt4* and their control strains (WT, Δ*pmt1::pmt1* and Δ*pmt4::pmt4*) in the following experiments of three replicates.

For all the fungal strains, 1 μl aliquots of 10^6^ conidia/ml suspensions were centrally spotted on rich SDAY and minimal CZA plates or on 1/SDAY alone (control) or supplemented with NaCl (0.8 M), H_2_O_2_ (4 mM) and Congo red (80 μg/ml), respectively. After 7 days of incubation at 25 °C and 12:12 h, the mean diameter of each colony was estimated as an index of growth rate using two measurements taken perpendicular to each other across the center of the colony on a given medium.

Conidiation capacity of each strain was quantified over the days of cultivation at 25 °C and 12:12 h on SDAY plates, which were spread with 100 μl aliquots of a 10^7^ conidia/ml suspension for culture initiation. From day 4 onwards, three culture plugs (5 mm diameter) were taken daily from each plate using a cork borer until conidial yield reached a peak in the WT culture on day 8. Conidia on each plug were released into 1 ml of 0.02% Tween 80 via 10 min vibration. Conidial concentration in the suspension was determined using a haemocytometer and converted to the number of conidia per cm^2^ culture.

The virulence of each strain against *G. mellonella* larvae (~300 mg *per capita*) was bioassayed with its conidia being topically applied for normal cuticle infection or injected into haemocoel for cuticle-bypassing infection. Cohorts of 50 larvae were immersed in 40 ml of a 10^7^ conidia/ml suspension (treatment) or 0.02% Tween 80 (control) for 10 s and then transferred onto towel paper for removal of excessive water. Alternatively, 5 μl of a 10^5^ conidia/ml suspension (treatment) or 0.02% Tween 80 (control) was injected into the haemocoel of each larva in each cohort. All of the treated cohorts were maintained in Petri dishes (15 cm diameter) at 25 °C and monitored at 12 h interval for mortality records. The resultant time-mortality trends were subjected to probit analysis, yielding LT_50_ as an index of virulence for each strain against *G. mellonella*.

All phenotypic data were subjected to one-factor analysis of variance, followed by Tukey’s honestly significant difference (HSD) test for the means among the tested strains.

## Additional Information

**How to cite this article**: Wang, Z.-K. *et al*. Global insight into lysine acetylation events and their links to biological aspects in *Beauveria bassiana*, a fungal insect pathogen. *Sci. Rep.* **7**, 44360; doi: 10.1038/srep44360 (2017).

**Publisher's note:** Springer Nature remains neutral with regard to jurisdictional claims in published maps and institutional affiliations.

## Supplementary Material

Supplementary Tables and Figure S1

## Figures and Tables

**Table 1:** Survey of Kac proteins and Kac sites in lysine acetylomes across organisms.

Organisms	Materials	Counts*			References
		Proteins	Kac proteins	Kac sites	
Prokaryotes					
*Thermus thermophilus*	cell culture	2173	128 (5.9)	197 (1.3)	16
*Escherichia coli*	cell culture	4931	91 (1.8)	138 (1.5)	17
	cell culture	4931	349 (7.1)	1070 (3.1)	18
*Salmonella enterica*	cell culture	4438	1901 (42.8)	235 (0.1)	19
*Erwinia amylovora*	cell culture	3315	96 (2.9)	141 (1.5)	49
*Vibrio parahemolyticus*	cell culture	4449	656 (14.7)	1413 (2.2)	22
*Mycobacterium tuberculosis*	cell culture	3906	137 (3.5)	226 (1.6)	21
*Bacillus subtilis*	cell culture	4175	185 (4.4)	332 (1.8)	20
*Streptomyces roseosporus*	cell culture	6807	667 (9.8)	1143 (1.7)	23
Eukaryotes					
*B. bassiana*	cell culture	10364	283 (2.7)	464 (1.6)	This study
*Saccharomyces cerevisiae*	cell culture	5907	1059 (19.6)	2878 (2.7)	24
*Candida albicans*	cell culture	9038	477 (5.3)	1073 (2.2)	25
*Phytophthora sojae*	cell culture	26489	1150 (4.3)	2197 (1.9)	26
*Arabidopsis thaliana*	mixed tissues	35378	57 (0.2)	64 (1.1)	28
	cell culture	35378	74 (0.2)	91 (1.2)	27
*Oryza sativa*	NB2P cells	36376	44 (0.1)	60 (1.4)	52
*Triticum aestivum*	leaves	99386	277 (0.3)	416 (1.5)	29
*Glycine max*	seeds	56044	121 (0.2)	190 (1.6)	53
*Vitis vinifera*	exo/mesocarp	26346	97 (0.4)	138 (1.4)	30
*Toxoplasma gondii*	tachyzoites	9255	274 (3.3)	411 (1.5)	31
*Plasmodium falciparum*	schizonts	5992	230 (3.8)	421 (1.8)	54
*Bombyx mori*	larvae/pupae	19618	342 (1.7)	667 (2.0)	32
*Drosophila melanogaster*	SL2 cells	30443	1013 (3.3)	1981 (2.0)	33
*Mus musculus*	liver cells	54152	1357 (2.5)	4067 (3.0)	55
*Rattus norvegicus*	16 tissues	38722	4541 (11.7)	15474 (3.4)	35
*Homo sapiens*	HeLa cells	71340	1348 (1.9)	2886 (2.1)	55
	liver cells		1042	1300 (1.2)	2,36

^*^Parenthesized values following the counts of columns 2 and 3 are percentages of Kac proteins in total proteins and means of Kac sites per Kac protein, respectively.
