# Supplementary material for: Global Insight into Lysine Acetylation Events and Their Links to Biological Aspects in Beauveria bassiana, a Fungal Insect Pathogen
Source: Sci Rep. 2017 Mar 15;7:44360. doi: 10.1038/srep44360 (PMC5353618; doi:10.1038/srep44360)
Supplement: Supplementary Tables and Figure S1 [file srep44360-s1.pdf]

Supporting Information for Scientific Reports

Global Insight into Lysine Acetylation Events and Their Links to Biological Aspects in *Beauveria bassiana,* a Fungal Insect Pathogen

Zhi-Kang Wang[1,2], Qing Cai[1], Jin Liu[1], Sheng-Hua Ying[1] & Ming-Guang Feng[1,]*

[1] Institute of Microbiology, College of Life Sciences, Zhejiang University, Hangzhou 310058, China (*Corresponding author at E-mail: mgfeng@zju.edu.cn)

[2] Shandong Provincial Key Laboratory of Microbial Engineering, Qilu University of Technology, Jinan, Shandong, 250353, China

Table S1. A list of Kac proteins and Kac sites identified from *B. bassiana* acetylome.

Protein accession	Kac-site position	Protein description	Score	Mass error [ppm]	Charge	m/z	Motif logo	Subcellular location
J4UI12	K-70	14-3-3 protein	90.79	-0.2628	2	716.9		cyto_nucl
J4UI12	K-78	14-3-3 protein	60.64	-1.5184	3	379.9	..........KH.........	cyto_nucl
J4UI12	K-124	14-3-3 protein	60.65	-0.1948	2	497.8		cyto_nucl
J4W7X9	K-263	1-aminocyclopropane-1-carboxylate synthase	69.70	-0.0546	3	866.1	........F.K..........	mito
J5K4S2	K-133	1-pyrroline-5-carboxylate dehydrogenase	60.55	2.3713	2	750.9	........F.K..........	mito
J5K4S2	K-435	1-pyrroline-5-carboxylate dehydrogenase	72.59	1.8995	3	791.1	........F.K..........	mito
J4KNY0	K-208	26S proteasome regulatory complex, ATPase RPT4	78.44	0.1537	2	868.5	..........KY.........	cyto
J4UGV5	K-89	26S proteasome regulatory subunit RPN5	118.87	0.3012	2	598.3	..........K....K.....	nucl
J4UGV5	K-203	26S proteasome regulatory subunit RPN5	63.22	-2.9550	2	505.3	..........KY.........	nucl
J4UGV5	K-261	26S proteasome regulatory subunit RPN5	119.74	1.9494	3	454.9	..........KY.........	nucl
J4UNI7	K-473	26S proteasome subunit RPN7	76.68	1.2972	2	552.8	..........KY.........	cyto
J4UHT4	K-313	2OG-Fe(II) oxygenase superfamily protein	57.43	-0.0153	2	786.9	..........KF.........	cysk
J4KMW7	K-729	3',5'-bisphosphate nucleotidase	54.00	-0.4156	3	596.0		mito
J4KPP6	K-59	40S ribosomal protein S0	101.97	0.7924	2	614.9		cyto
J4W087	K-75	40S ribosomal protein S1	90.39	0.0382	3	715.0		nucl
J4W087	K-109	40S ribosomal protein S1	88.28	-0.7585	3	685.0		nucl
J4UUU0	K-95	40S ribosomal protein S11	90.61	1.5904	2	633.8		mito
J5K2F7	K-79	40S ribosomal protein S2	70.55	0.4407	2	553.8	..........K....K.....	cyto
J4WKD9	K-88	40S ribosomal protein S22	62.47	-1.6746	2	650.4	..........KW........	mito
J4WKD9	K-92	40S ribosomal protein S22	90.71	0.2878	2	562.4		mito
J5JNL6	K-167	40S ribosomal protein S7	78.52	-0.2516	2	522.3		mito
J4ULU5	K-162	40S ribosomal protein S8	156.81	0.2925	2	781.9		nucl
J4VYD0	K-113	40S ribosomal protein S9	72.29	2.2238	2	718.4	..........K....K.....	mito
J4VYD0	K-152	40S ribosomal protein S9	122.94	-1.7468	3	711.0	..........KH.........	mito
J4W0F2	K-421	4-aminobutyrate transaminase GatA	52.01	0.2785	2	783.4	........F.K..........	mito
J4UJD4	K-48	60S ribosomal protein L12	99.99	0.4139	2	716.4		cyto
J5JBW1	K-42	60S ribosomal protein L14-B	148.28	-0.4103	3	469.3		cyto
J5JBW1	K-98	60S ribosomal protein L14-B	99.44	0.2603	2	509.8	..........KW........	cyto
J5JDJ3	K-84	60S ribosomal protein L16	107.49	0.0349	2	469.8	........F.K..........	mito
J5JDJ3	K-150	60S ribosomal protein L16	48.90	0.7865	3	610.3	..........KY.........	mito
J5JMG2	K-356	60S ribosomal protein L18	118.01	0.6777	2	473.8		mito
J5K377	K-92	60S ribosomal protein L20	89.36	-0.6258	3	476.6		cyto_nucl
J5K377	K-131	60S ribosomal protein L20	88.02	0.4344	2	643.8	..........K....K.....	cyto_nucl
J4KRI8	K-47	60S ribosomal protein L28	75.09	0.4141	3	527.9	..........KY.........	mito
J4KRI8	K-77	60S ribosomal protein L28	77.22	-1.9066	3	665.0		mito
J5J6J0	K-115	60S ribosomal protein L3	96.77	3.0165	3	643.0	..........K....K.....	mito
J5J6J0	K-120	60S ribosomal protein L3	64.76	1.2351	2	545.8	........F.K..........	mito
J4UI15	K-49	60S ribosomal protein L36	47.22	-0.6943	2	801.4		mito
J5K106	K-13	60S ribosomal protein L43	102.52	0.9510	2	540.3	..........KY.........	mito
J5K106	K-36	60S ribosomal protein L43	76.82	-0.2180	3	663.6	..........KY.........	mito
J4W3G8	K-43	60S ribosomal protein L5 (CPR4)	122.79	2.2721	2	477.2	..........KY.........	nucl
J4W3G8	K-271	60S ribosomal protein L5 (CPR4)	68.92	0.0845	2	432.8	..........KY.........	nucl
J5K698	K-26	60S ribosomal protein L6	74.99	-0.2129	2	762.3	..........KW.........	mito
J5JR46	K-203	60S ribosomal protein L7	80.01	0.4545	2	588.3		cyto
J5JR46	K-227	60S ribosomal protein L7	68.37	1.3685	2	518.3	..........K....K.....	cyto
J5JR46	K-302	60S ribosomal protein L7	69.35	-1.0305	3	516.6	..........KH.........	cyto
J4UTA8	K-260	6-phosphogluconate dehydrogenase, decarboxylating	78.34	-0.3048	2	674.8	..........KW........	cyto
J4UTA8	K-414	6-phosphogluconate dehydrogenase, decarboxylating	120.57	1.2530	2	713.4	........F.K..........	cyto
J4UTA8	K-437	6-phosphogluconate dehydrogenase, decarboxylating	66.89	0.1274	3	764.7	........F.K..........	cyto
J4UKH5	K-187	ABC transporter	48.29	-0.3563	3	666.7		cyto
J4UKH5	K-355	ABC transporter	87.22	0.8006	2	779.4	..........KY.........	cyto
J4UKH5	K-561	ABC transporter	77.74	2.1467	2	467.3		cyto
J4UKH5	K-1024	ABC transporter	55.11	3.7366	2	509.8		cyto
J4VXX6	K-1549	ABC transporter	56.02	-2.7396	2	1108.5		plas
J5JKE7	K-161	Acetyl-CoA acetyltransferase	115.80	-0.5363	2	860.4	..........KY.........	cyto
J5JKE7	K-255	Acetyl-CoA acetyltransferase	82.43	-0.3413	2	508.3		cyto
J5JYH3	K-267	Acetyl-CoA acetyltransferase	87.15	0.3183	2	585.4		mito

J4VT77	K-344	Acidic chitinase	81.71	-0.3860	2	726.3	..........KY.........	cyto
J4VT77	K-349	Acidic chitinase	53.57	2.4791	2	670.8	..........KY.........	cyto
J4VS61	K-612	Aconitate hydratase	132.24	-0.5990	3	640.3	..........KY.........	mito
J4UN10	K-84	Actin-like protein	96.32	0.4191	5	695.1		cysk
J4UN10	K-315	Actin-like protein	94.49	-0.6124	2	803.9		cysk
J4ULX7	K-88	Acyl CoA binding protein	99.11	0.6552	2	572.3	..........KY.........	nucl
J4W9D6	K-102	Adenosine kinase	107.35	-2.4283	3	533.6		cyto
J4W508	K-207	Adenosine/AMP deaminase	68.54	-1.2306	2	601.8	..........KW........	cyto
J5JW61	K-39	Adenosylhomocysteinase	107.17	0.4933	2	715.9	..........KY.........	cyto
J5JW61	K-145	Adenosylhomocysteinase	82.89	1.6190	3	908.8	..........KY.........	cyto
J5JW61	K-151	Adenosylhomocysteinase	134.66	-0.2432	4	731.8		cyto
J5JW61	K-191	Adenosylhomocysteinase	135.86	1.3657	2	651.3	..........KF.........	cyto
J5JW61	K-325	Adenosylhomocysteinase	135.47	-0.9854	2	806.4		cyto
J4UIB1	K-368	Adenylate-forming enzyme AfeA	85.68	0.5461	2	509.3	........F.K..........	cyto
J5JFV8	K-50	ADP, ATP carrier protein	157.86	0.8643	2	843.9		mito
J5JFV8	K-256	ADP, ATP carrier protein	90.61	-1.1737	2	709.3	..........KY.........	mito
J4KQQ4	K-36	ADP-ribosylation factor 6	91.85	0.1208	2	511.3		mito
J4KQQ4	K-11	ADP-ribosylation factor family protein	45.74	1.7391	2	524.8	........F.K..........	mito
J4WGE9	K-362	Aldehyde dehydrogenase	52.72	1.3921	3	758.7		cyto
J4UG93	K-691	Amidohydrolase-like protein	50.22	-0.6003	2	631.4	..........KY.........	cyto
J5JKW5	K-687	Aminopeptidase 2	70.69	-0.3844	2	639.3	..........K....K.....	cyto
J4WML8	K-247	Annexin	78.32	-0.7202	2	513.8	..........K....K.....	nucl
J5JB33	K-446	ANTH domain-containing protein	62.46	-0.6253	2	483.3	..........KY.........	nucl
J4UVV7	K-274	Arp2/3 complex	48.57	0.4680	3	484.2		nucl
J5JGQ2	K-366	Aspartate aminotransferase, putative	110.08	-0.9537	2	641.9	..........KY.........	cyto_nucl
J4KL14	K-9	ATP synthase subunit D	58.13	-0.6538	2	580.8		mito
J5K5W4	K-91	ATP synthase subunit g	84.73	-0.0721	2	786.5		mito
J4KLN7	K-175	ATP-citrate synthase subunit 1	64.00	-0.3427	2	921.0	..........K....K.....	cyto_mito
J4KLN7	K-497	ATP-citrate synthase subunit 1	101.65	-1.2938	2	545.3	........F.K..........	cyto_mito
J4KLN7	K-542	ATP-citrate synthase subunit 1	63.47	1.3989	2	680.9	..........KF.........	cyto_mito
J4WC21	K-35	Calcofluor white hypersensitive protein	57.18	1.1201	3	477.6	..........KF.........	cyto
J4KKZ9	K-113	Calreticulin family protein	51.77	-0.9888	3	510.9	..........KF.........	cyto
J4KKZ9	K-433	Calreticulin family protein	45.61	0.4845	3	509.3	..........KH.........	cyto
J4UT30	K-538	Carbamoyl-phosphate synthase arginine-specific large chain	47.33	0.4617	2	555.8		mito
J4ULC5	K-273	Catalase-like domain, heme-dependent	76.04	1.0005	2	533.8	..........KW........	extr
J4VRQ4	K-421	Catalase-peroxidase	89.51	-1.7768	3	558.0	........F.K..........	extr
J4VRQ4	K-771	Catalase-peroxidase	62.11	0.3586	2	455.8	........F.K..........	extr
J4VRQ4	K-779	Catalase-peroxidase	52.25	1.3733	2	645.8		extr
J4KPM3	K-60	CgERG6-2 protein	118.19	-0.4512	3	623.3		cyto
J5JJ97	K-410	Chaperonin GroL	77.05	0.3983	2	538.8	..........KF.........	mito
J5JJ97	K-468	Chaperonin GroL	79.15	2.2170	2	532.3	..........KF.........	mito
J5J6H0	K-70	Citrate synthase	162.37	-0.0089	2	831.4		mito
J5J6H0	K-345	Citrate synthase	90.05	-0.8567	2	640.8		mito
J5JDL3	K-711	Clathrin heavy chain	77.66	0.2317	2	605.8	..........KY.........	cyto_nucl
J4KKQ2	K-364	Coatomer subunit beta	67.02	-2.4554	2	755.9		cyto
J4KQF4	K-128	Coatomer WD associated region	50.19	-0.6757	2	645.3		cyto
J5J4V7	K-458	Cobalamin-independent methionine synthase	126.09	0.4393	2	786.9	........F.K..........	cyto
J5J4V7	K-472	Cobalamin-independent methionine synthase	87.18	1.1443	2	710.4		cyto
J5J4V7	K-547	Cobalamin-independent methionine synthase	67.25	-0.2221	2	627.8	..........KY.........	cyto
J5J4V7	K-726	Cobalamin-independent methionine synthase	40.53	-0.4482	3	910.5	........F.K..........	cyto
J4KQJ3	K-280	Coenzyme A transferase	85.36	-0.3342	2	586.3	..........KY.........	mito
J4KLB3	K-22	Coiled-coil domain-containing protein	68.54	-1.3368	2	646.8	..........KF.........	nucl
J4WIM3	K-89	Copper transport protein ctr4	117.79	-0.5239	2	695.4	..........KF.........	plas
J5JXQ5	K-262	Coproporphyrinogen III oxidase	73.93	0.0356	3	552.6		mito
J5K731	K-550	Cse1-like protein	124.07	-0.4038	2	763.4		cyto_nucl
J4KR46	K-77	Ctr copper transporter	77.66	2.0447	2	561.8		plas
J4UIE0	K-39	Cyanide hydratase	131.83	0.2996	2	637.8		cyto
J5JU37	K-49	Cyclophilin A	136.02	-0.2306	2	642.8		cyto
J5JU37	K-66	Cyclophilin A	148.41	-0.2797	2	915.9	..........KF.........	cyto
J5JU37	K-115	Cyclophilin A	92.94	1.9612	3	561.0	........F.K..........	cyto
J4UQW6	K-328	Cyclophilin type peptidyl-prolyl cis-trans isomerase/CLD	109.07	-0.4471	3	553.6		cyto

J4KQY5	K-440	Cyclopropane-fatty-acyl-phospholipid synthase	137.95	2.7791	2	582.8	..........KY.........	plas
J4KNY5	K-394	Cys/Met metabolism PLP-dependent enzyme	94.78	-2.8508	2	767.4		mito
J4UHI8	K-303	Cystathionine beta-synthase	71.50	1.1316	2	485.3	..........K....K.....	cyto
J4KPU1	K-126	Cytochrome b-c1 complex subunit Rieske, mitochondrial	110.61	-3.2388	2	485.8	..........KW........	mito
J4ULH7	K-82	Cytochrome c	80.32	1.3153	2	712.9		mito
J4UT43	K-92	Cytochrome C1 family protein	91.31	0.3996	3	497.6		mito
J5JYR9	K-462	Cytochrome oxidase assembly protein	79.12	1.3290	2	436.3		plas
J5JYL6	K-350	Cytochrome P450 CYP6001C8	99.50	0.8860	2	721.4	..........KY.........	mito
J5JYL6	K-711	Cytochrome P450 CYP6001C8	63.98	-1.1321	4	705.9	..........KH.........	mito
J5JYL6	K-769	Cytochrome P450 CYP6001C8	63.19	0.4822	2	808.4		mito
J4WA82	K-206	Cytochrome P450 CYP625A1	44.58	-0.5662	4	581.8		extr
J4KLW5	K-144	Cytoplasmic ribosomal protein subunit S3	115.00	-0.1057	2	623.3		mito
J4WBV9	K-46	DDHD domain-containing protein	91.70	1.5224	2	604.8	..........K....K.....	extr
J4UK32	K-30	Dienelactone hydrolase	83.86	1.0381	2	586.3	..........KY.........	cyto
J5JA60	K-168	Dipeptidyl peptidase	108.43	-0.3302	2	572.3		mito
J5JA60	K-606	Dipeptidyl peptidase	82.42	-0.4975	2	675.3	..........KW........	mito
J5JA60	K-664	Dipeptidyl peptidase	65.18	0.9079	4	686.4		mito
J5JHB1	K-5	Dopa 4,5-dioxygenase	115.91	-0.2489	2	743.4		mito
J5JI32	K-27	DUF543 domain-containing protein	131.66	-0.2335	2	814.9	..........KW........	extr
J4WLY4	K-109	DUTP diphosphatase	112.36	-0.1887	3	745.7	..........KH.........	cyto
J5JAL2	K-601	Efflux pump antibiotic resistance protein	92.27	0.5039	3	600.3		plas
J4UPD5	K-163	eIF4A-like protein	79.89	2.3531	2	506.3	........F.K..........	nucl
J4UPD5	K-229	eIF4A-like protein	96.23	0.7934	2	594.3		nucl
J4UPD5	K-282	eIF4A-like protein	93.62	0.0812	2	680.4		nucl
J5K2R4	K-37	Elongation factor 1-alpha	212.90	-0.6701	3	650.3	..........K....K.....	cyto
J5K2R4	K-42	Elongation factor 1-alpha	84.57	-0.3450	2	468.8	..........KF.........	cyto
J5K2R4	K-63	Elongation factor 1-alpha	63.43	-1.6532	2	589.3		cyto
J5K2R4	K-85	Elongation factor 1-alpha	93.23	-0.4992	3	670.3	..........KY.........	cyto
J5K2R4	K-160	Elongation factor 1-alpha	156.48	-2.3764	2	633.8	..........KW........	cyto
J5K2R4	K-171	Elongation factor 1-alpha	59.71	0.0497	2	814.4		cyto
J5K2R4	K-225	Elongation factor 1-alpha	105.02	1.7979	3	609.0		cyto
J5K2R4	K-254	Elongation factor 1-alpha	114.90	-0.0319	2	1012.6		cyto
J5K2R4	K-377	Elongation factor 1-alpha	122.69	2.5719	2	646.3		cyto
J5K2R4	K-407	Elongation factor 1-alpha	75.53	-1.5252	2	1169.1		cyto
J5JKW9	K-86	Elongation factor 1-gamma	117.89	0.0223	2	452.3		cyto
J5JKW9	K-143	Elongation factor 1-gamma	142.94	-1.1904	3	735.7	..........KY.........	cyto
J4KQT0	K-293	Elongation factor 2	89.47	-0.3065	3	769.1		cyto
J4KQT0	K-322	Elongation factor 2	65.47	1.3819	2	499.8		cyto
J4KQT0	K-430	Elongation factor 2	95.78	0.2063	2	637.9	........F.K..........	cyto
J5JYW0	K-92	Emp24/gp25L/p24 family/GOLD	62.86	-0.5470	3	590.3	..........KY.........	extr
J5JFU0	K-29	Ethyl tert-butyl ether degradation ethD	58.59	-0.0034	3	465.6	..........KH.........	cyto
J4UM55	K-261	Eukaryotic initiation factor 4E	125.72	0.3418	2	648.3	..........KW........	cyto
J5JMF8	K-313	Eukaryotic translation initiation factor 3 subunit L	74.53	-1.0670	2	568.3	..........KY.........	nucl
J5JE25	K-71	Eukaryotic translation initiation factor 5A	74.78	0.7895	3	744.4	..........KY.........	cyto
J4KQ38	K-108	Farnesyl pyrophosphate synthetase	90.99	-1.1523	2	504.7		cyto
J4KRH9	K-159	Fasciclin domain-containing protein	79.84	-0.8349	4	482.8	..........KF.........	extr
J5JE67	K-980	Fatty acid synthase subunit alpha	75.79	0.9684	2	649.4		cyto
J5JE67	K-1067	Fatty acid synthase subunit alpha	58.32	1.2038	3	511.9	..........KF.........	cyto
J5JE67	K-1168	Fatty acid synthase subunit alpha	94.09	-1.0401	2	777.4		cyto
J4WHG6	K-504	F-box domain-containing protein	41.45	2.3143	3	380.6		cyto
J4UQG5	K-243	Fe-containing alcohol dehydrogenase	64.52	-1.8049	2	877.5		mito
J5JWL1	K-79	Feruloyl esterase A	89.91	-0.1532	2	905.9	..........K....K.....	extr
J4KR90	K-339	FK506-binding protein	66.83	-1.6492	2	866.9	........F.K..........	cyto
J4KQ04	K-203	FMN-binding split barrel-related protein	68.02	-3.0007	2	533.8		cyto
J4KQ04	K-209	FMN-binding split barrel-related protein	107.46	0.0396	2	695.3	..........KH.........	cyto
J4KQ04	K-214	FMN-binding split barrel-related protein	93.50	-0.2265	4	623.0	..........KY.........	cyto
J4KQ04	K-264	FMN-binding split barrel-related protein	73.25	0.7119	2	537.8		cyto
J4KRF1	K-128	Gamma interferon inducible lysosomal thiol reductase (GILT)	72.34	-2.1682	3	722.7	..........KH.........	nucl
J4W919	K-183	Glucanosyltransferase-like protein	87.48	2.8534	2	551.8	........F.K..........	extr
J5K3W9	K-148	Glucosamine 6-phosphate acetyltransferase, putative	222.94	-0.6785	3	600.6		cyto
J4UI24	K-155	Glucose-6-phosphate 1-dehydrogenase	59.15	-0.6575	2	529.8		cyto_nucl

J4UI24	K-347	Glucose-6-phosphate 1-dehydrogenase	84.69	-1.9523	2	622.3		cyto_nucl
J4UTF9	K-132	Glucose-6-phosphate isomerase	75.14	2.6320	3	632.6	..........KH.........	cyto
J4UTF9	K-310	Glucose-6-phosphate isomerase	74.99	0.3152	3	510.9	..........KH.........	cyto
J4UTF9	K-313	Glucose-6-phosphate isomerase	91.87	-0.2605	2	521.3	..........K....K.....	cyto
J5K727	K-198	Glutamate dehydrogenase	53.58	-0.9038	3	750.7	..........KH.........	cyto
J5K727	K-231	Glutamate dehydrogenase	79.66	-2.0397	2	587.3	..........KW........	cyto
J5K727	K-454	Glutamate dehydrogenase	88.09	-2.4620	2	817.4		cyto
J4UUX9	K-291	Glutamine synthetase	157.57	-1.2374	3	856.7		mito
J5JMH9	K-131	Glutamine:fructose-6-phosphate amidotransferase	65.16	-3.4835	2	546.8		cyto
J5JMH9	K-149	Glutamine:fructose-6-phosphate amidotransferase	81.71	-0.6159	2	713.4	..........KY.........	cyto
J5K3E1	K-130	Glutathione peroxidase	75.74	-0.4893	2	503.8	..........KW........	cyto
J5K3E1	K-135	Glutathione peroxidase	112.13	0.0047	2	669.4	..........KF.........	cyto
J4VV24	K-29	Glutathione reductase	57.79	-0.0120	2	527.8	..........KF.........	cyto
J4VYF5	K-369	Glutathione S-transferase	61.42	1.4406	2	693.9	..........KW........	mito
J5JP22	K-99	Glutathione S-transferase II	53.38	0.5764	3	522.6	..........KW........	nucl
J4KL75	K-228	Glutathione-dependent formaldehyde dehydrogenase	110.97	0.0193	2	893.0	..........K....K.....	cyto
J4KLJ6	K-27	Glutathione-S-transferase theta, GST	87.68	-0.5342	3	687.4		cyto
J4KLJ6	K-47	Glutathione-S-transferase theta, GST	78.94	-0.4956	2	574.8	..........KF.........	cyto
J4KLJ6	K-205	Glutathione-S-transferase theta, GST	89.91	-1.1908	3	779.7		cyto
J5JSE5	K-48	Glyceraldehyde-3-phosphate dehydrogenase	133.48	-0.7136	2	968.5	..........KY.........	cyto
J5JSE5	K-167	Glyceraldehyde-3-phosphate dehydrogenase	148.20	0.2504	3	911.1	..........KF.........	cyto
J5JSE5	K-194	Glyceraldehyde-3-phosphate dehydrogenase	72.29	3.0482	2	637.3		cyto
J5JSE5	K-259	Glyceraldehyde-3-phosphate dehydrogenase	78.90	1.0450	2	625.3		cyto
J5JSE5	K-333	Glyceraldehyde-3-phosphate dehydrogenase	84.51	0.6451	2	832.0		cyto
J5JM30	K-196	Glycolipid transfer protein	117.20	-0.0198	2	701.9		cyto
J5J5Z7	K-213	Glycoside hydrolase, family 47	88.02	-0.0233	2	674.9	..........KY.........	extr
J5J5Z7	K-277	Glycoside hydrolase, family 47	51.73	-1.4268	2	848.9		extr
J4UQ14	K-536	Glycosyl hydrolase family 2	95.64	-0.0577	2	557.3		extr
J5JB66	K-88	Glycosyltransferase family 39	51.14	1.4645	3	510.3	..........KF.........	plas
J5JB66	K-482	Glycosyltransferase family 39	73.62	3.4726	2	468.8	..........KF.........	plas
J5K1X2	K-360	Glycosyltransferase family 39	108.72	1.0886	3	557.6	..........KY.........	plas
J5JE04	K-54	GNAT family acetyltransferase, putative	57.24	-1.1303	3	921.8		nucl
J4UW03	K-136	Gp24-like protein	109.07	0.2672	2	868.9	..........KW........	extr
J4UGD8	K-70	GPI anchored protein, putative	114.86	0.6077	2	720.9	..........KW........	extr
J4KM02	K-157	GTP-binding nuclear protein GSP1/Ran	113.25	1.4097	2	914.0	........F.K..........	cyto
J4KQZ2	K-41	GTP-binding protein SAR1	74.70	3.5235	2	692.4		cyto
J5J4Q5	K-261	H3 K56 histone acetylation protein KAT11	128.38	0.4292	2	510.2		mito
J5JE18	K-250	Heat shock protein	60.20	0.6466	3	494.2	..........KY.........	cyto
J4W4F4	K-57	Heat shock protein 70-2	61.36	-1.2248	3	799.4		cyto
J4W4F4	K-72	Heat shock protein 70-2	84.51	-1.3813	3	628.6		cyto
J4W4F4	K-89	Heat shock protein 70-2	105.14	-0.4179	3	655.6	..........KH.........	cyto
J4W4F4	K-94	Heat shock protein 70-2	92.54	1.7684	2	586.8		cyto
J4W4F4	K-247	Heat shock protein 70-2	185.25	-1.2569	2	715.9	..........K....K.....	cyto
J4W4F4	K-526	Heat shock protein 70-2	61.66	0.8780	3	576.3	..........KY.........	cyto
J4W4F4	K-591	Heat shock protein 70-2	74.03	-0.5191	3	891.8		cyto
J4UK77	K-177	Heat shock protein Hsp90	79.34	0.6096	3	767.4		nucl
J4UK77	K-333	Heat shock protein Hsp90	108.46	0.5974	2	531.3		nucl
J4UK77	K-378	Heat shock protein Hsp90	54.07	-0.5460	2	637.8		nucl
J5JXN3	K-521	Heat shock protein HSP98	93.35	0.4213	2	716.4	..........KY.........	cyto_nucl
J5K3S2	K-32	Hemolysin-III protein	84.51	1.8459	2	635.8	..........KY.........	plas
J5JID8	K-112	Hexokinase-like protein	110.08	-0.2107	2	714.4	..........KY.........	cyto
J5JID8	K-323	Hexokinase-like protein	43.46	-2.0078	4	666.8	..........KH.........	cyto
J5K0S3	K-237	Histidyl-tRNA synthetase	66.27	-1.4122	2	533.3		cyto
J4UF78	K-6	Histone H2A	162.76	0.2125	2	952.0		nucl
J4UF78	K-10	Histone H2A	162.76	0.2125	2	952.0		nucl
J4UF78	K-15	Histone H2A	162.76	0.2125	2	952.0		nucl
J4WER3	K-5	Histone H2A	173.81	0.9469	3	768.0		nucl
J4WER3	K-7	Histone H2A	173.81	0.9469	3	768.0		nucl
J4WER3	K-12	Histone H2A	173.81	0.9469	3	768.0	..........K....K.....	nucl
J4WER3	K-17	Histone H2A	173.81	0.9469	3	768.0		nucl
J5J2Q8	K-9	Histone H2B	164.59	-0.4435	3	556.0		nucl

J5J2Q8	K-13	Histone H2B	127.91	0.4081	2	769.4		nucl
J5J2Q8	K-20	Histone H2B	164.59	0.4081	2	769.4	..........K....K.....	nucl
J5J2Q8	K-119	Histone H2B	77.22	0.3249	3	602.3	..........KH.........	nucl
J4VQS0	K-19	Histone H3	132.01	0.4953	2	528.8	..........K....K.....	nucl
J4VQS0	K-24	Histone H3	132.01	0.4953	2	528.8		nucl
J4VQS0	K-57	Histone H3	123.79	0.0859	2	646.9		nucl
J4VQS0	K-80	Histone H3	125.72	-1.2505	2	682.3		nucl
J5JXL7	K-6	Histone H4	157.20	-0.1098	3	480.3		nucl
J5JXL7	K-9	Histone H4	157.20	-0.1098	3	480.3		nucl
J5JXL7	K-13	Histone H4	157.20	-0.1098	3	480.3		nucl
J5JXL7	K-17	Histone H4	157.20	-0.1098	3	480.3		nucl
J4UWE3	K-352	Hsp70-like protein	65.30	0.2119	2	704.9		cyto
J4W1P7	K-262	Hsp70-like protein	105.46	0.3740	2	630.3	..........K....K.....	mito
J5JYV0	K-126	Hsp70-like protein	111.79	-0.5378	2	768.9	..........KH.........	extr
J4UQZ0	K-114	Hsp90 associated co-chaperone	103.88	2.4959	3	435.9	........F.K..........	cyto
J5K1F3	K-5	Hydroxyisourate hydrolase	169.67	-0.1825	2	1035.0		cyto
J5JRR7	K-37	Hydroxymethylglutaryl-CoA synthase	58.02	-0.8319	2	884.5	..........KY.........	cyto
J4W2G6	K-253	Ice nucleation protein	70.20	1.6569	2	721.3		extr
J4UIH1	K-312	Importin-beta domain-containing protein	74.99	-1.2461	3	532.3	..........KW........	mito
J4UFJ6	K-182	Inorganic pyrophosphatase	51.73	-1.6459	2	784.5		mito
J5K833	K-512	Isocitrate lyase	59.17	-0.8913	3	615.6	..........KW........	cyto
J4ULY0	K-211	Isopentenyl-diphosphate delta-isomerase	53.03	1.2246	2	680.4		cyto_nucl
J4UL04	K-886	Karyopherin Kap123	73.25	-0.5506	2	508.3	........F.K..........	cyto
J4UMM7	K-78	Ketol-acid reductoisomerase	54.90	0.2611	2	854.9		mito
J4UMM7	K-258	Ketol-acid reductoisomerase	93.91	-2.4123	2	939.9	........F.K..........	mito
J4KML5	K-285	La domain-containing protein	85.81	0.0199	2	712.3		cyto_nucl
J4KN99	K-63	L-PSP endoribonuclease family protein Brt1	82.84	0.8001	2	815.5		cyto
J4KPU2	K-205	LysM domain-containing protein	51.29	-0.8270	2	672.8	..........KF.........	extr
J4URT3	K-145	LysM domain-containing protein	102.65	-0.7550	2	866.9	..........KY.........	extr
J4URT3	K-206	LysM domain-containing protein	87.32	-1.0247	2	535.2	..........KW........	extr
J4URT3	K-369	LysM domain-containing protein	75.91	-0.2480	2	791.4		extr
J4KMZ7	K-69	Malate dehydrogenase	101.05	1.3976	2	919.0		extr
J4KMZ7	K-201	Malate dehydrogenase	79.68	-0.3295	2	993.5	..........KY.........	extr
J5K3K1	K-446	Malic enzyme	67.10	0.5750	4	472.0		cyto
J4W2E4	K-18	Mannitol dehydrogenase	123.79	1.2390	2	564.4		cyto
J4W2E4	K-68	Mannitol dehydrogenase	134.99	0.5255	2	690.9	..........K....K.....	cyto
J5J9C2	K-155	Membrane-associated progesterone receptor component 1	143.50	-2.5233	2	628.3	..........KY.........	extr
J5J5R7	K-504	Metallopeptidase family M24	56.51	-0.4675	3	599.6	..........KF.........	mito
J4W7A9	K-334	Methyltransferase-like protein	74.48	0.1598	2	831.0		extr
J4W7A9	K-377	Methyltransferase-like protein	115.38	-0.6506	2	952.5		extr
J4WD26	K-95	Mitochondrial 2-oxodicarboxylate carrier 1	95.42	-0.6512	2	773.4	..........KF.........	mito
J4UW62	K-91	Mitochondrial ATPase inhibitor, putative	65.51	-0.4017	3	614.0		mito
J5JJY0	K-305	Mitochondrial DNA replication protein YHM2	68.54	1.9814	2	598.3	..........K....K.....	cyto
J5JI80	K-20	Mitochondrial genome maintenance protein Mgr2	88.95	-0.1374	3	756.0	..........KF.........	plas
J5K9Z1	K-177	Mitochondrial peroxiredoxin PRX1	138.45	-0.7345	3	475.6	..........KH.........	cyto
J5K9Z1	K-214	Mitochondrial peroxiredoxin PRX1	74.42	0.8299	2	465.8		cyto
J5J2F8	K-164	Mitochondrial RNA-splicing protein MRS3	42.03	-2.4853	2	724.3	..........KY.........	mito
J5K2U1	K-414	Mitochondrial-processing peptidase subunit beta	138.91	-0.1917	3	711.4		mito
J5JK39	K-130	Mitogen-activated protein kinase	77.19	1.2926	3	488.9	..........KY.........	nucl
J4KN09	K-3	Monooxygenase-like protein	90.73	-0.8117	3	738.8		extr
J4UHJ1	K-127	Monooxygenase-like protein	77.74	0.5075	2	515.3		extr
J4UHJ1	K-238	Monooxygenase-like protein	67.90	-1.3627	2	528.8	..........KW........	extr
J4KNP1	K-1152	Multidrug resistance protein CDR1	89.30	0.1736	2	658.3		plas
J5K4T1	K-200	NAD dependent epimerase/dehydratase	62.17	0.9860	3	425.9	..........KH.........	cyto
J4UQW0	K-67	NADH dehydrogenase iron-sulfur protein	60.16	0.3883	2	736.9	..........KY.........	mito
J4KPK3	K-307	NADH-ubiquinone oxidoreductase 51 kDa subunit	44.85	-0.3974	3	442.6	..........KH.........	mito
J5JZP4	K-60	NADH-ubiquinone oxidoreductase B18 subunit	54.01	-0.5578	3	553.9		nucl
J5JZM7	K-194	NADPH dehydrogenase	88.02	-1.1154	2	645.8	..........KW........	mito
J5JZM7	K-291	NADPH dehydrogenase	58.92	-0.7797	2	484.8	..........KY.........	mito
J4UN70	K-57	NADPH--cytochrome P450 reductase	91.96	-2.0553	2	705.8		cyto
J4VS23	K-59	Nascent polypeptide-associated complex (NAC) subunit	101.97	-1.5169	3	374.9		nucl

J5JPV3	K-19	Outer mitochondrial membrane protein porin	137.86	-0.3419	3	828.4		cyto
J5JPV3	K-112	Outer mitochondrial membrane protein porin	82.75	-2.0355	2	540.3	..........KF.........	cyto
J5JPV3	K-118	Outer mitochondrial membrane protein porin	113.42	0.2049	3	535.0		cyto
J5JPV3	K-224	Outer mitochondrial membrane protein porin	148.41	1.1211	2	753.9	..........KY.........	cyto
J4WL44	K-393	Oxidoreductase molybdopterin binding domain-containing protein	74.42	1.4659	2	497.3	..........KW........	pero
J4WLK3	K-142	Oxidoreductase NAD-binding domain-containing protein	50.18	-0.3480	2	908.0	..........KY.........	mito
J4WLK3	K-201	Oxidoreductase NAD-binding domain-containing protein	76.33	-1.4924	2	708.4	..........KY.........	mito
J5K7I8	K-272	Oxysterol-binding protein	48.82	-1.1306	3	508.6	..........KY.........	mito
J4KRA6	K-242	PA domain-containing protein	112.71	0.6555	2	618.9	..........KY.........	plas
J4KRA6	K-815	PA domain-containing protein	137.95	-0.6993	2	644.8		plas
J5JQG2	K-347	PAN domain containing protein	101.89	-0.5062	2	712.8		extr
J5JQG2	K-529	PAN domain containing protein	94.45	-0.3025	3	528.6	..........K....K.....	extr
J4KNM4	K-308	Peptidase family M1	102.40	0.8556	2	668.8		cyto_nucl
J4KNM4	K-438	Peptidase family M1	83.18	1.4689	2	671.9	..........K....K.....	cyto_nucl
J4UIY7	K-401	Peptidase family S58	124.38	-0.0644	2	654.9		mito
J4VZK5	K-301	Peptidase S1 and S6, chymotrypsin/Hap	102.98	-0.5289	2	1246.6	..........KY.........	extr
J4VZK5	K-397	Peptidase S1 and S6, chymotrypsin/Hap	110.41	0.5153	2	567.8	..........KY.........	extr
J4WH14	K-42	Peptidyl-prolyl cis-trans isomerase	92.87	-0.5815	2	554.8	..........KF.........	cyto
J4UR82	K-335	PH domain-containing protein	70.09	-2.7157	2	761.4		cyto_nucl
J4VZ63	K-210	Phosphate carrier protein 2	117.03	1.3289	2	785.9	........F.K..........	mito
J4WIT3	K-83	Phosphatidylinositol/phosphatidylglycerol transfer protein	96.67	0.1977	2	545.8	..........KY.........	cyto
J4UR50	K-204	Phosphoenolpyruvate carboxykinase	94.49	0.0236	2	938.4	..........KY.........	cyto
J4W4J4	K-48	Phospholipid methyltransferase	126.31	-0.3404	2	580.8		extr
J4KR41	K-208	Phosphomannomutase	80.17	2.0014	3	516.9	..........KH.........	cyto
J4KR41	K-231	Phosphomannomutase	107.12	1.2001	3	440.6		cyto
J4VUB7	K-434	Plasma membrane ATPase (Proton pump)	52.79	-0.3314	2	424.8	........F.K..........	plas
J4VUB7	K-450	Plasma membrane ATPase (Proton pump)	52.01	-0.8062	3	578.6		plas
J4KNF7	K-31	Plectin/S10 domain-containing protein	148.57	0.7101	2	786.4	..........KH.........	mito
J4KNF7	K-44	Plectin/S10 domain-containing protein	98.68	1.6545	2	840.0		mito
J4UKQ9	K-101	Proteasome A-type and B-type	98.65	-0.5006	2	831.4	..........KY.........	cyto
J4KLY7	K-56	Proteasome component PUP3	122.79	0.9176	2	508.3		extr
J4WHE2	K-296	Proteasome regulatory subunit 12	101.20	0.5266	3	642.3		cyto
J5JHT2	K-101	Protein disulfide isomerase	83.31	0.1295	3	994.1		E.R.
J4UJA2	K-21	Protein yop-1	66.00	-0.0303	2	794.9	..........KY.........	plas
J5JN27	K-134	Putative aldehyde dehydrogenase	98.11	-1.3478	2	733.9		cyto
J5JN27	K-313	Putative aldehyde dehydrogenase	113.47	0.1870	3	663.7	..........KF.........	cyto
J5JN27	K-343	Putative aldehyde dehydrogenase	81.45	-0.1811	2	854.9		cyto
J5JN27	K-373	Putative aldehyde dehydrogenase	99.97	0.0508	2	545.8	..........K....K.....	cyto
J5JN27	K-378	Putative aldehyde dehydrogenase	73.89	-0.6243	2	784.4	........F.K..........	cyto
J4W097	K-254	Putative enolase	89.48	-1.4442	2	945.4	........F.K..........	cyto
J4W097	K-259	Putative enolase	64.27	0.4813	2	485.7	..........KY.........	cyto
J4W097	K-347	Putative enolase	124.08	0.7875	3	833.5		cyto
J4W097	K-410	Putative enolase	76.22	1.3033	2	555.9		cyto
J4UUV2	K-12	Putative MLH1 protein	75.89	3.1305	2	767.9		cyto_nucl
J5JEV5	K-36	Putative peroxisomal membrane protein	128.95	-1.6862	3	861.7	..........KY.........	cyto
J5JEV5	K-118	Putative peroxisomal membrane protein	66.47	-1.5873	3	843.4	..........KF.........	cyto
J5JEV5	K-121	Putative peroxisomal membrane protein	84.75	-2.2860	2	777.4	........F.K..........	cyto
J4KN49	K-109	Putative phospholipase	109.10	-0.9673	3	557.6		extr
J5K3I9	K-26	Putative Rab7-like GTPase	76.33	-4.6008	2	627.8	..........K....K.....	mito
J5JKJ0	K-233	Putative serine/threonine phosphatase 2C ptc2	99.57	0.4796	2	557.8	..........K....K.....	cyto
J4UFB0	K-124	Putative succinyl-CoA ligase	98.75	1.1840	2	572.3		mito
J5JU37	K-31	Putative U-snRNP-associated cyclophilin	84.48	-2.7380	2	659.3		cyto
J4KNG5	K-56	Putative YHM1 (Mitochondrial carrier)	65.81	-0.1411	2	744.9		cyto
J4KNG5	K-92	Putative YHM1 (Mitochondrial carrier)	113.93	-0.4494	2	647.4	..........KY.........	cyto
J5JW20	K-343	Pyruvate dehydrogenase E1 component subunit alpha	59.12	0.6969	2	661.9	..........KH.........	mito
J5JW20	K-345	Pyruvate dehydrogenase E1 component subunit alpha	74.92	-0.2184	2	491.3	..........K....K.....	mito
J5JW20	K-358	Pyruvate dehydrogenase E1 component subunit alpha	117.53	0.0355	2	559.8		mito
J5K3K3	K-488	Pyruvate kinase	108.47	-0.2165	2	665.8		mito
J4UN61	K-173	Ras-like protein	60.35	-0.7678	2	737.4	..........KF.........	cysk
J5JWA1	K-136	Replication factor C	48.09	-0.0881	2	604.3	..........KY.........	cyto
J5JMS9	K-22	Ribosomal family S4e	100.97	1.1344	3	708.7		mito

J5JMS9	K-53	Ribosomal family S4e	119.29	0.0041	2	541.8	..........KY.........	mito
J5JMS9	K-106	Ribosomal family S4e	127.56	0.6719	2	504.3		mito
J4W316	K-27	Ribosomal L28e family protein	66.56	-0.1657	3	435.9		nucl
J4W316	K-58	Ribosomal L28e family protein	58.28	0.0752	3	598.7		nucl
J4W316	K-89	Ribosomal L28e family protein	89.90	-1.2775	2	583.3	........F.K..........	nucl
J4KPZ3	K-93	Ribosomal protein L15	122.39	-0.4725	2	795.9	..........KY.........	mito
J4KPZ3	K-157	Ribosomal protein L15	50.35	-0.2929	4	389.5	..........KH.........	mito
J4KMN0	K-2823	Ribosomal protein L19	69.49	0.0480	3	613.3	..........KH.........	cyto
J4KMN0	K-2834	Ribosomal protein L19	51.73	1.1360	4	508.0	..........K....K.....	cyto
J4KMN0	K-2862	Ribosomal protein L19	70.55	-1.6275	2	531.8		cyto
J5K134	K-141	Ribosomal protein L7Ae	52.19	0.3731	4	554.3	..........KY.........	cyto
J5K134	K-221	Ribosomal protein L7Ae	65.23	1.8751	2	623.9		cyto
J5K134	K-233	Ribosomal protein L7Ae	134.99	-0.2442	3	454.5	..........KH.........	cyto
J5J7W5	K-60	Ribosomal protein S7	96.23	-0.1550	3	511.9	..........KY.........	cyto
J4UPN1	K-94	Ribosomal protein S9/S16	107.74	-0.0181	3	619.3	..........KF.........	mito
J4UPN1	K-105	Ribosomal protein S9/S16	120.87	-1.4819	2	715.4		mito
J4VQS5	K-114	RNA recognition domain-containing protein	79.15	-0.7903	3	437.9		nucl
J5JHE5	K-350	RNA recognition domain-containing protein	68.02	0.1785	2	509.3	..........K....K.....	nucl
J5JL84	K-158	RNA recognition domain-containing protein	73.44	-2.0859	2	666.4	..........KY.........	nucl
J4KNV3	K-97	Saccharopine dehydrogenase [NAD(+), L-lysine-forming]	45.42	-1.1713	5	533.5		cyto
J5JFA5	K-316	Sarcosine oxidase	110.08	-0.6384	2	652.9	..........KY.........	extr
J5JHW2	K-338	Serine/threonine protein kinase	67.65	0.7032	3	660.7		cyto
J5JX00	K-308	Serine/threonine-protein kinase gad8	59.07	-1.8827	2	575.8	........F.K..........	mito
J4KN65	K-141	Serine/threonine-protein phosphatase	99.57	-0.8332	2	696.8		nucl
J5JPN4	K-10	Spermidine synthase	103.76	1.7698	2	885.9		cyto
J5JPN4	K-40	Spermidine synthase	89.17	-0.1862	2	634.8	..........KY.........	cyto
J5JPN4	K-151	Spermidine synthase	56.34	-0.3619	3	628.3	..........KF.........	cyto
J5JPN4	K-268	Spermidine synthase	76.07	0.5884	2	789.3		cyto
J5JG47	K-150	Steroid monooxygenase (CpmA), putative	99.50	2.0907	2	631.3	..........KW........	cyto
J5JG47	K-186	Steroid monooxygenase (CpmA), putative	52.58	0.5945	2	738.9	..........KF.........	cyto
J5JG47	K-300	Steroid monooxygenase (CpmA), putative	100.11	-0.5898	2	886.4		cyto
J5JG47	K-495	Steroid monooxygenase (CpmA), putative	84.48	2.5139	2	691.9	..........KY.........	cyto
J5JSD1	K-544	Stress protein ORP150	73.83	-0.1337	2	864.9	..........KY.........	E.R.
J5JM98	K-211	Sublitisin-like protease	118.57	-0.0386	4	772.6	..........KW........	cyto
J5JM98	K-253	Sublitisin-like protease	76.23	2.2575	2	587.9		cyto
J5JBK8	K-162	Sulphydryl oxidase	77.22	0.5459	3	794.8	..........K....K.....	extr
J5JT33	K-197	T-complex protein 1 subunit alpha	71.69	-1.3515	2	509.8	..........KY.........	cyto
J5K8Q8	K-68	TH14-3-3 protein	58.32	0.7236	2	716.9		cyto_nucl
J5K8Q8	K-82	TH14-3-3 protein	74.85	1.5613	2	768.9	..........K....K.....	cyto_nucl
J5K8Q8	K-105	TH14-3-3 protein	92.24	-0.4415	3	707.7	..........KH.........	cyto_nucl
J5K8Q8	K-117	TH14-3-3 protein	64.06	-1.9659	3	408.5	..........K....K.....	cyto_nucl
J5K8Q8	K-122	TH14-3-3 protein	60.65	-0.1948	2	497.8		cyto_nucl
J5K289	K-64	Thioredoxin-like protein	67.75	-2.4110	2	646.3	........F.K..........	cyto
J4KPZ5	K-121	Transaldolase	71.35	-0.0117	2	880.5		cyto
J4KPZ5	K-189	Transaldolase	77.06	1.3980	2	625.8		cyto
J5JRZ3	K-99	Transaldolase-like protein	86.14	0.5404	2	472.3		mito
J4WBF7	K-91	Transketolase	110.08	1.7210	2	638.8		cyto
J4WBF7	K-241	Transketolase	68.79	1.3043	2	421.8	..........K....K.....	cyto
J4WBF7	K-246	Transketolase	61.16	-1.4542	2	556.3		cyto
J4WBF7	K-318	Transketolase	125.61	-0.6756	3	749.0	..........KY.........	cyto
J4WBF7	K-392	Transketolase	75.82	1.6177	2	638.3		cyto
J5JHY6	K-102	Triosephosphate isomerase	72.32	0.3242	2	737.9		cyto
J5JHY6	K-144	Triosephosphate isomerase	68.97	-0.8066	2	714.4		cyto
J5JHY6	K-185	Triosephosphate isomerase	100.11	0.0932	3	575.0		cyto
J5JA67	K-309	tRNA synthetase class II core domain-containing protein	85.27	-3.0575	3	550.9	..........KY.........	cyto
J5JVN5	K-107	Ubiquinol-cytochrome C reductase	84.48	0.7399	2	703.3		nucl
J4UG75	K-6	Ubiquitin subgroup	81.92	0.0886	2	654.4		nucl
J5K530	K-415	Ubiquitin-activating enzyme E1	72.34	1.4383	2	651.3		cyto
J4KP06	K-38	Uncharacterized protein	97.73	-1.6802	2	640.3		extr
J4KPL0	K-33	Uncharacterized protein	153.39	1.3478	2	799.3		extr
J4KR02	K-62	Uncharacterized protein	66.22	-0.6822	2	697.9		extr

J4UEX9	K-262	Uncharacterized protein	92.93	1.1074	3	760.4		extr
J4UHZ3	K-191	Uncharacterized protein	42.34	-1.8898	2	700.8	..........KY.........	extr
J4UI57	K-209	Uncharacterized protein	94.26	0.9370	2	453.8		plas
J4UI95	K-209	Uncharacterized protein	65.37	-1.1240	4	489.5		extr
J4UK64	K-457	Uncharacterized protein	68.54	2.7300	3	465.9		extr
J4UK64	K-463	Uncharacterized protein	77.74	1.0684	2	675.9		extr
J4UNC3	K-499	Uncharacterized protein	62.46	0.6882	3	378.2	..........KY.........	nucl
J4UQ40	K-133	Uncharacterized protein	124.38	0.7474	2	732.4		cysk
J4URP7	K-81	Uncharacterized protein	89.36	-1.5550	2	544.8	..........K....K.....	cyto
J4URP7	K-116	Uncharacterized protein	61.24	-0.7827	2	651.8		cyto
J4URP7	K-128	Uncharacterized protein	78.35	-0.1238	3	948.1		cyto
J4USU4	K-89	Uncharacterized protein	95.27	-0.7966	2	698.4	..........KY.........	mito
J4VTX0	K-67	Uncharacterized protein	107.65	-0.4941	3	812.3	..........KH.........	extr
J4W895	K-44	Uncharacterized protein	146.59	-0.1168	2	910.9		cyto
J4WGT0	K-247	Uncharacterized protein	77.90	-0.3750	3	779.4		mito
J4WGT0	K-254	Uncharacterized protein	69.91	0.9396	3	362.6		mito
J4WGT0	K-269	Uncharacterized protein	73.30	-0.6155	2	963.5		mito
J4WGT0	K-329	Uncharacterized protein	101.05	0.1150	2	949.5	..........KY.........	mito
J5J6U1	K-81	Uncharacterized protein	92.04	1.0491	2	803.9	..........KY.........	mito
J5JIY6	K-112	Uncharacterized protein	66.58	-0.0899	4	991.7	..........KH.........	extr
J5JIY6	K-151	Uncharacterized protein	117.00	-1.2091	3	605.7	..........KW........	extr
J5JIY6	K-200	Uncharacterized protein	93.11	0.1491	3	577.9		extr
J5JIY6	K-209	Uncharacterized protein	139.48	0.8786	2	749.8		extr
J4UF76	K-201	Uroporphyrinogen decarboxylase	75.74	4.2723	2	714.4	..........KY.........	cyto
J5JA74	K-331	UTP-glucose-1-phosphate uridylyltransferase	121.68	0.0273	3	420.2		cyto_nucl
J4KMP5	K-603	Vacuolar ATP synthase catalytic subunit A	54.02	0.9796	2	709.9	..........KF.........	mito
J5K2B5	K-316	Valyl-tRNA synthetase	90.61	1.5703	2	652.9		cyto
J5K2B5	K-514	Valyl-tRNA synthetase	95.09	-0.0216	3	508.9		cyto
J4UL34	K-195	Vesicular integral-membrane protein VIP36	89.17	1.2399	2	694.9	..........KY.........	extr
J5JEH5	K-130	WD domain-containing protein	100.22	0.1824	2	745.9		nucl
J5JEH5	K-139	WD domain-containing protein	100.68	0.1689	2	935.5	..........KY.........	nucl
J5JEH5	K-145	WD domain-containing protein	106.30	-0.2311	3	665.3		nucl
J5JEH5	K-172	WD domain-containing protein	69.77	0.8216	3	752.1		nucl
J5JEH5	K-175	WD domain-containing protein	67.88	0.3235	2	695.9		nucl
J4UFI9	K-457	Woronin body major protein	64.80	-0.1418	3	499.6		mito_nucl
J4KN64	K-71	WSC domain-containing protein	91.55	0.6506	2	671.3		extr
J4KN64	K-662	WSC domain-containing protein	98.94	-0.7522	3	638.9	..........KH........	extr
J4KN64	K-667	WSC domain-containing protein	111.64	-0.4833	2	996.4		extr
J5J2K5	K-169	Xaa-Pro aminopeptidase, putative	75.82	-0.3512	3	439.6	..........KY........	cyto
J4KQV4	K-288	Zinc-binding dehydrogenase	61.82	-0.0454	3	708.3	..........KY........	cyto
J5JZ31	K-253	Zinc-binding dehydrogenase	78.19	1.0346	2	487.8	..........K....K....	cyto

Table S2. GO distribution of identified lysine acetylated proteins (IKacPs) in *B. bassiana*.

GO Terms Level 1	GO Terms Level 2	No. IKacPs
Biological Process	metabolic process	179
	cellular process	141
	single-organism process	40
	localization	28
	biological regulation	15
	response to stimulus	14
	cellular component organization or biogenesis	10
	other	5
	unclassification	77
Cellular Component	cell	84
	organelle	60
	macromolecular complex	55
	membrane	34
	other	2
Molecular Function	catalytic activity	148
	binding	135
	structural molecule activity	32
	transporter activity	15
	electron carrier activity	7
	other	8
	unclassification	51

Table S3. Predicted subcellular localization of identified lysine acetylated proteins (IKacPs) in *B. bassiana*.

Subcellular Location	No. IKacPs
Cytosol (Cyto)	105
Mitochondria (Mito)	68
Extracellular space (Extr)	40
Nucleus (Nucl)	32
Plasma membrane (Plas)	15
Cytosol and nucleus (Cyto_Nucl)	14
Cytoskeleton (Cysk)	4
Endoplasmic reticulum (ER)	2
Cytosol and mitochondria (Cyto_Mito)	1
Mitochondria and nucleus (Mito_Nucl)	1
Peroxisome (Pero)	1

Table S4. FunCat annotation of identified lysine acetylated proteins (IKacPs) in *B. bassiana*.

Functional category	No. IKacPs
Biogenesis of cellular components	41
Cell cycle and dna processing	25
Cell fate	19
Cell rescue, defense and virulence	52
Cell type differentiation	14
Cellular communication/signal transduction mechanism	15
Development	1
Energy	48
Interaction with the environment	25
Metabolism	114
Protein fate	59
Protein synthesis	46
Protein with binding function or cofactor requirement	143
Regulation of metabolism and protein function	17
Systemic interaction with the environment	2
Transcription	25
Transposable elements, viral and plasmid proteins	1

Table S5. KOG classification of identified lysine acetylated proteins (IKacPs) in *B. bassiana*.

KOG Classification	No. KacPs
INFORMATION STORAGE AND PROCESSING	**51**
[J] Translation, ribosomal structure and biogenesis	42
[K] Transcription	2
[L] Replication, recombination and repair	2
[B] Chromatin structure and dynamics	5
CELLULAR PROCESSES AND SIGNALING	**72**
[Y] Nuclear structure	3
[T] Signal transduction mechanisms	8
[M] Cell wall/membrane/envelope biogenesis	3
[Z] Cytoskeleton	3
[W] Extracellular structures	1
[U] Intracellular trafficking, secretion, and vesicular transport	13
[O] Posttranslational modification, protein turnover, chaperones	41
METABOLISM	**106**
[C] Energy production and conversion	35
[G] Carbohydrate transport and metabolism	19
[E] Amino acid transport and metabolism	16
[F] Nucleotide transport and metabolism	3
[H] Coenzyme transport and metabolism	5
[I] Lipid transport and metabolism	12
[P] Inorganic ion transport and metabolism	7
[Q] Secondary metabolites biosynthesis, transport and catabolism	9
POORLY CHARACTERIZED	**26**
[R] General function prediction only	23
[S] Function unknown	3

Table S6. Paired primers used for Kac site mutations of *pmt1* and *pmt4* in *B. bassiana* via overlap extension PCR.

Primers	Sequences *	Purposes
K88R-F	CGCTTCCAAGTACATCAAGGGACGTTTTTTCATGGATGTTCACCC	Cloning mutant *pmt1*^{K88R}
K88R-R	GGGTGAACATCCATGAAAAAACGTCCCTTGATGTACTTGGAAGCG	Cloning mutant *pmt1*^{K88R}
K482R-F	GTCTCCGTACCATCGAGACCCGTTTCCGTCTCGTTCATCTCAT	Cloning mutant *pmt1*^{K482R}
K482R-R	ATGAGATGAACGAGACGGAAACGGGTCTCGATGGTACGGAGAC	Cloning mutant *pmt1*^{K482R}
K360R-F	ACCTCCACAGCCATGATGAGCGTTACCCTCTCCGCTACGAAGA	Cloning mutant *pmt4*^{K360R}
K360R-R	TCTTCGTAGCGGAGAGGGTAACGCTCATCATGGCTGTGGAGGT	Cloning mutant *pmt4*^{K360R}
sK88R-F	ACGGAGTCGAGCGTTATT	Sequencing mutant *pmt1*^{K88R}
sK88R-R	TTTCGGCGTTACTTGTTG	Sequencing mutant *pmt1*^{K88R}
sK482R-F	ACGGAGTCGAGCGTTATT	Sequencing mutant *pmt1*^{K482R}
sK482R-R	TTTCGGCGTTACTTGTTG	Sequencing mutant *pmt1*^{K482R}
sK360R-F	CATTCCATTGCGGTGCTG	Sequencing mutant *pmt4*^{K360R}
sK360R-R	GATGCCTGGCTTGGTTCA	Sequencing mutant *pmt4*^{K360R}
dK88R-F	CTGCTTTCTTGCGGACTCGT	PCR detecting Δ*pmt1*/*pmt1*^{K88R}
dK88R-R	GCCGTAGGCTTACTGGACTT	PCR detecting Δ*pmt1*/*pmt1*^{K88R}
dK482R-F	CTGCTTTCTTGCGGACTCGT	PCR detecting Δ*pmt1*/*pmt1*^{K482R}
dK482R-R	GCCGTAGGCTTACTGGACTT	PCR detecting Δ*pmt1*/*pmt1*^{K482R}
dK360R-F	ACAATCTCCCTTCTCCTGCT	PCR detecting Δ*pmt4*/*pmt4*^{K360R}
dK360R-R	GCACCTTGCCAAAGTGAACT	PCR detecting Δ*pmt4*/ *pmt4*^{K360R}
qPmt1-F/R	GCAACACATTCTACGACAA / AACTCAACCTTCTCTTCCTT	qRT-PCR detecting *pmt1*
qPmt4-F/R	TCGCCTCTCCTTATTATCC / ACTGTCTTGAACTCTTGGT	qRT-PCR detecting *Pmt4*
18S-F/R	TGGTTTCTAGGACCGCCGTAA / CCTTGGCAAATGCTTTCGC	qRT-PCR detecting 18S RNA

* The red region of each primer denotes the coding sequence for the mutation of each Kac site (K residue) to R residue.

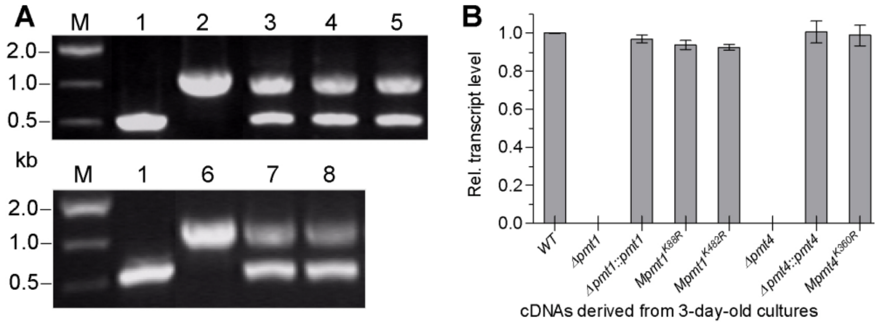

Figure S1 Identifying Kac site-mutated strains of *B. bassiana* Pmt1 and Pmt4. (**A**) PCR detection of *pmt1* and *pmt4* in the cDNAs derived from 3-day-old cultures of WT (lane 1), Δ*pmt1* (lane 2), Δ*pmt1*::*pmt1* (lane 3), M*pmt1*^{K88R} (lane 4), M*pmt1*^{K482R} (lane 5), Δ*pmt4* (lane 6), Δ*pmt4*::*pmt4* (lane 7), and M*pmt4*^{K360R} (lane 8), respectively. (**B**) Transcript levels of *pmt1* in the cultures of *pmt1* mutants and of *pmt4* in the cultures of *pmt4* mutants with respect to the WT standard. Note that a singular Kac site mutation had no effect on the expression of *pmt1* or *pmt4*. Error bars: SD from three cDNA samples.
